# Photo- and Immunotherapy Interface: Can Dendritic Cell Vaccines Overcome the Limitations of PDT?

**DOI:** 10.3390/pharmaceutics18050588

**Published:** 2026-05-10

**Authors:** Natalia Shilyagina, Yevgeniya Sannova, Victoria Turubanova, Irina Balalaeva

**Affiliations:** 1 Institute of Biology and Biomedicine, Lobachevsky State University of Nizhny Novgorod, Nizhny Novgorod 603022, Russia; 2 Department of Genetics and Life Sciences, Sirius University, Sochi 354340, Russia; 3 Institute of Neurosciences, Lobachevsky State University, Nizhny Novgorod 603022, Russia

**Keywords:** photosensitizer, immunogenic cell death, apoptosis, necroptosis, ferroptosis, pyroptosis, DAMPs, PDT resistance, antigen presentation, antitumor effect

## Abstract

Photodynamic therapy (PDT) occupies an important place in the arsenal of cancer treatment modalities; however, its efficacy is primarily limited by the local nature of its effects and by tumor cell resistance. The aim of this review is to analyze the fundamental principles and biological consequences of PDT, to summarize current data on the molecular and cellular mechanisms determining its efficacy, and to consider strategies for overcoming its limitations. Particular attention is paid to the mechanisms underlying resistance development and to the role of switching from non-immunogenic to immunogenic cell death in shaping the antitumor response. The potential integration of PDT with dendritic cell vaccination is considered a promising strategy for overcoming these limitations. The potential of vaccine-based approaches to activate specific antitumor immunity in aggressive cancers is highlighted, with emphasis on the advantages of dendritic cell vaccines in addressing the limitations of conventional PDT.

## 1. Introduction: The Principle of Photodynamic Therapy

Photodynamic therapy (PDT) is a minimally invasive, organ-preserving treatment modality used for cancer, skin diseases, and certain infectious diseases of bacterial, viral, and fungal origin, as well as in dentistry and ophthalmology. PDT is based on the application of photoactive dyes—photosensitizers (PS) [1,2,3].

The photodynamic effect was first described in 1900 by O. Raab, a student of H. von Tappeiner, at the University of Munich [1,2,3,4,5]. These researchers demonstrated that *Paramecium* sp., when incubated with acridine and other fluorescent dyes, died upon subsequent exposure to light, whereas they remained viable when exposed to light alone or when incubated with the dye in the absence of light. H. von Tappeiner termed this phenomenon “photodynamic,” referring to the effect of light on cellular dynamics, i.e., mobility. He subsequently introduced the term “photodynamic reaction” to describe a specific photochemical process leading to molecular destruction in the presence of light, a photosensitizer, and oxygen [6]. The application of photodynamic reactions in oncology dates back to 1903, when H. von Tappeiner, together with A. Jesionik, reported the first successful treatment of skin cancer using eosin in combination with sunlight. However, photodynamic therapy in its modern form originates from the work of S. Schwartz and R. Lipson, who in the 1960s demonstrated that mixtures of hematoporphyrin derivatives (HpD) selectively accumulate in tumors following intravenous administration and can be visualized intraoperatively due to their characteristic red fluorescence [7]. PDT is currently approved and actively used for cancer treatment in several leading regions, including North America (the largest and most developed market, with the United States as the leader), Europe (notably Germany, France, and the United Kingdom), and the Asia-Pacific region (China, India, Japan, and South Korea) (based on materials from the Strategic Market Research portal, https://www.strategicmarketresearch.com/market-report/cancer-photodynamic-therapy-market, accessed on 27 April 2026). Two recent reviews [8,9] have made a substantial contribution to the systematization of current knowledge on PDT by providing a detailed analysis of its efficacy and clinical applications across various tumor types. In the present review, we focus on the beneficial antitumor properties of PDT and examine how their combination with immunotherapy may help overcome its limitations.

PDT involves the administration of a photoactive compound, or photosensitizer, which, upon activation by light of a specific wavelength (typically ~650–850 nm) in the presence of tissue oxygen, induces the generation of highly cytotoxic reactive oxygen species (ROS). The mechanism underlying the photodynamic effect has been described in detail [1,10,11] and proceeds as follows: upon absorption of a photon, the PS molecule transitions to an excited singlet state (^1^PS*), followed by intersystem crossing to a long-lived triplet state (^3^PS*), from which it can participate in several types of photochemical reactions. The most common are type I and type II photochemical reactions involving molecular oxygen. In type I reactions, ^3^PS* participates in redox processes, transferring an electron either to dissolved O_2_ or to organic molecules. In the latter case, the resulting organic radicals or radical ions subsequently react with oxygen to form reduction products. As a result, organic radicals and various ROS accumulate in the medium, including the superoxide anion radical (O_2_^−^•), hydroperoxyl radical (HO_2_•), hydrogen peroxide (H_2_O_2_), and hydroxyl radical (OH•). In type II reactions, ^3^PS* directly transfers energy to molecular oxygen via triplet–triplet interaction, resulting in the formation of highly reactive singlet oxygen (^1^O_2_) [1,12,13]. In practice, most photosensitizers are capable of inducing both type I and type II reactions, and the balance between these pathways depends primarily on the chemical structure of the compound and the concentration of dissolved oxygen. A decrease in the partial pressure of O_2_ increases the contribution of type I reactions [14,15]. This shift alters the spectrum of ROS generated during the photodynamic reaction, which consumes oxygen and may lead to hypoxia in the irradiated tissue.

In addition to type I and II reactions, oxygen-independent mechanisms of the photodynamic effect, characteristic of certain classes of photosensitizers, have attracted increasing attention in recent years. The concept of type III photosensitizers is based on their ability, following light absorption, to selectively bind to various macromolecules, such as nucleic acids and proteins, and to inactivate them specifically. Studies investigating the mechanisms of type III reactions remain limited [14,16,17]. A representative example is the formation of DNA cross-links mediated by photoactivated psoralen. It is also important to note the photoisomerization of drug molecules, as exemplified by combretastatin, which produces a light-mediated therapeutic effect and is sometimes classified as a type IV photochemical reaction [18]. In brief, such photosensitizers are initially unable to bind to their molecular targets and undergo intramolecular rearrangement (photoisomerization) upon light excitation, which subsequently enables binding to cellular targets. Notably, type III and IV reactions are effective under hypoxic conditions, where type I and II photosensitization is limited [11].

The accumulation of ROS and organic radicals within the cell initiates free-radical oxidation of lipids, proteins, and nucleic acids. The formation of significant amounts of oxidized fatty acid derivatives alters membrane fluidity and viscosity, leading to increased permeability due to the appearance of polar groups in acyl residues and disruption of the ordered bilayer structure [19,20,21]. Protein oxidation results in the accumulation of atypical oxidized groups, primarily carbonyl groups, in amino acid side chains, leading to loss of native protein conformation. This disrupts the function of transport proteins, enzymes, and macromolecular complexes, and alters protein–protein interactions in signaling and regulatory pathways [22,23]. The formation of intermolecular cross-links (protein–protein, protein–lipid, or lipid–lipid) is also possible, facilitated by the accumulation of dialdehydes generated during oxidative degradation of lipid acyl chains, as well as by the formation of radical derivatives of cystine and cysteine in proteins. Oxidative stress additionally leads to damage to nucleic acids, including oxidative modification of nitrogenous bases (most prominently guanine), formation of apurinic/apyrimidinic sites, and cleavage of phosphodiester bonds in the sugar–phosphate backbone [24].

The effectiveness of PDT largely depends on the intensity of ROS generation; however, the ultimate outcome of oxidative stress is determined by the capacity of the cellular antioxidant system [25,26]. Cellular protection is provided by a combination of low-molecular-weight compounds (glutathione, ubiquinone/coenzyme Q10, vitamins C and E, alpha-lipoic acid, and thioredoxins), present in both aqueous and membrane phases, as well as by enzymatic systems (superoxide dismutase, catalase, glutathione peroxidase, and glutathione reductase). These systems function to neutralize ROS and to restore or eliminate oxidized biomolecules [26]. Several studies have demonstrated that oxidative stress induced by PDT can activate transcription factors such as NF-κB (Nuclear factor kappa B), AP-1 (Activator protein 1), and HIF-1 (Hypoxia inducible factor 1), as well as early response genes (c-fos, c-jun), which regulate the expression of proteins involved in cell survival, apoptosis, and anti-inflammatory responses [27,28,29]. The therapeutic strategy of PDT is to ensure ROS generation at levels that exceed the capacity of cellular defense systems and are therefore incompatible with cell survival.

Since the discovery of the photodynamic effect, numerous compounds with photosensitizing properties and potential applications in PDT have been synthesized or isolated from natural sources. Currently, three generations of such compounds are used in clinical practice. First-generation photosensitizers include agents based on hematoporphyrin derivatives (natural tetrapyrrole compounds) [30]. Second-generation photosensitizers comprise modified macrocyclic tetrapyrrole compounds, such as chlorins, bacteriochlorins, and phthalocyanines, as well as 5-aminolevulinic acid, which serves as a precursor for the biosynthesis of endogenous protoporphyrin IX. These compounds are characterized by a high absorption coefficient in the far-red or near-infrared spectral range and an increased capacity to generate ROS, enabling their use in the treatment of larger and deeper tumors. The third generation consists of second-generation photosensitizers combined with carriers that enable targeted and selective delivery to tumor tissue (e.g., nanoparticles, liposomes, polymeric nanocapsules) or conjugated with targeting moieties such as monoclonal antibodies, peptides, and carbohydrates [31].

Despite the large number of compounds classified as photosensitizers that have been developed to date, only a limited number have reached clinical application (Supplementary Table S1). The structures of most clinically approved photosensitizers are based on tetrapyrrole frameworks; however, several non-macrocyclic compounds are also used, including hypericin, curcumin, toluidine blue, rose bengal, eosin, and methylene blue [32]. Among these, hypericin, an anthraquinone derivative isolated from *Hypericum perforatum* L., is one of the most well-known.

The initial decades of clinical experience with PDT led to the formulation of the concept of the so-called “ideal photosensitizer” [33,34]. According to these criteria, an ideal photosensitizer should absorb light within the optical transparency window of biological tissues (700–950 nm) [18]; selectively accumulate in tumor tissue while sparing healthy tissues; exhibit high photodynamic activity while remaining non-toxic in the absence of light; and be rapidly eliminated from the body. In addition, it should possess a stable and reproducible chemical composition, well-defined production methods, and low production costs [35]. These requirements, widely discussed in both scientific and clinical communities, have guided research in this field for many years.

One of the key limitations of PDT is its dependence on oxygen, as the process consumes molecular oxygen, and reduced oxygen levels significantly limit ROS generation. An alternative approach to cancer treatment is photothermal therapy (PTT), which employs specific substances (photothermal agents) that generate heat upon light exposure, particularly in the near-infrared range. The resulting increase in temperature in surrounding tissues leads to tumor cell destruction. The advantages of PTT include high precision, low invasiveness, oxygen independence, and favorable efficacy. However, similarly to PDT, PTT requires the development of imageable photothermal agents for accurate treatment planning and monitoring. Combined photodynamic and photothermal therapy is being extensively investigated as a strategy to enhance therapeutic efficacy [36,37,38]. PDT and PTT complement each other due to their distinct mechanisms of action and side-effect profiles. Although PTT also utilizes light (typically in the near-infrared range), it employs higher power densities to elevate tissue temperature and achieve local photocoagulation, resulting in rapid cell death through protein denaturation and membrane damage [36]. Despite certain technical similarities, important differences between PDT and PTT have significant clinical implications. PDT exhibits higher intrinsic selectivity due to the combined selectivity of photosensitizer accumulation and localized light irradiation. However, photosensitizer accumulation in normal tissues may lead to increased photosensitivity. In contrast, the selectivity of PTT is primarily determined by localized light delivery, which inevitably produces a temperature gradient. The absence of mechanisms for precise heat confinement can result in thermal damage beyond the targeted area, increasing the risk of adverse effects [37]. Another key difference lies in the requirements for light sources. PDT employs relatively low power densities and energy fluences, allowing the use of light-emitting diodes, low-power lasers, and even daylight for photosensitizer activation. In contrast, PTT requires high-energy laser systems, fiber-optic cooling, and real-time temperature monitoring.

Effective PDT depends on the presence of a functional vascular network and sufficient oxygen levels for ROS generation, whereas PTT is largely oxygen-independent and therefore applicable to hypoxic tumors [38]. Consequently, the combination of PDT and PTT appears promising, particularly for overcoming hypoxia in the tumor microenvironment [36]. The development of multifunctional nanoparticles that simultaneously act as photosensitizers and photothermal absorbers may further address the challenge of targeted delivery to tumor cells [39,40]. Moreover, unlike PDT, which requires intracellular accumulation of the photosensitizer, PTT is less dependent on the precise intracellular distribution of the photothermal agent, provided that the required temperature is achieved throughout the treated area.

Thus, it is now evident that no simple strategy exists for achieving maximal PDT efficacy. Addressing the challenge of harnessing the beneficial properties of PDT while overcoming its limitations has become a major focus of research. This requires consideration not only of the physicochemical properties of photosensitizers but also of the metabolic characteristics of tumor cells of different origins, their heterogeneity within a single tumor, potential off-target effects, and the role of the host organism in shaping the systemic response to PDT.

## 2. Molecular Mechanisms of PDT Resistance

One of the key stages in the development of PDT was the gradual recognition that, similarly to chemotherapy and radiotherapy, this treatment modality encounters tumor cell resistance, both intrinsic (primary) and acquired (adaptive). Primary resistance to PDT may arise for several reasons related to both tumor characteristics and treatment conditions. Thus, resistance in cells subjected to PDT may develop due to insufficient selectivity of PS accumulation within the tumor, rapid elimination of the PS from tumor tissue (a short half-life reduces the duration of exposure), and a low quantum yield of singlet oxygen generation. Technical factors include an excessively low dose rate or insufficient irradiation time to achieve adequate damage, as well as a suboptimal interval between PS administration and irradiation [41,42,43]. Tumor progression after PDT may be driven by multiple factors, including tumor hypoxia, tissue heterogeneity, high expression of antioxidant systems that neutralize ROS, and active DNA repair mechanisms.

One of the most extensively studied causes of resistance to PDT is tumor hypoxia [44]. During PDT, active oxygen consumption occurs, leading to the activation of a hypoxia-associated transcriptional program triggered by stabilization of the HIF-1α subunit and formation of the active HIF-1 transcription factor. HIF-1–dependent expression of vascular endothelial growth factor (VEGF) promotes angiogenesis. In addition, HIF-1–mediated signaling contributes to metabolic reprogramming of tumor cells, thereby enhancing their survival and proliferation [45,46]. Ultimately, this results in the development of a more aggressive malignant phenotype and increased resistance to therapy. Various approaches have been proposed to mitigate the negative effects of hypoxia during PDT, including hyperbaric oxygenation, the use of HIF-1 inhibitors, administration of exogenous oxygen carriers such as perfluorocarbons and hemoglobin, and the use of catalysts capable of generating O_2_ in situ, including photosynthetic bacteria and catalase [45,47,48,49].

Because PDT is based on the induction of pronounced oxidative stress, many resistance mechanisms are associated with enhanced antioxidant capacity. Tumor progression is accompanied by increased ROS production, elevated basal oxidative stress, and compensatory activation of antioxidant systems. Tumor cells with upregulated antioxidant defenses are therefore intrinsically less sensitive to PDT. This has been demonstrated for cells with increased expression of the Mn-dependent mitochondrial isoform of superoxide dismutase (MnSOD, SOD2) [50,51], the Cu,Zn-dependent cytosolic isoform (Cu,Zn-SOD, SOD1) [52], catalase [53], the phospholipid hydroperoxide-reducing isoform of glutathione peroxidase (GPX4) [54,55], and glutathione S-transferase (GST) [56]. Accordingly, strategies aimed at enhancing PDT efficacy have been proposed based on inhibition of glutathione (GSH) activity, including approaches involving catalytic conversion of hydrogen peroxide into intracellular oxygen using catalase or magnesium oxide [57].

A key transcriptional regulator maintaining the prooxidant–antioxidant balance in cells is nuclear factor erythroid 2-related factor 2 (Nrf2). Elevated ROS levels, including those induced by PDT, lead to inactivation of its inhibitor Keap1 (Kelch like ECH associated protein 1), enabling Nrf2 translocation into the nucleus and activation of transcription of a broad range of genes encoding antioxidant enzymes, as well as proteins involved in iron metabolism and xenobiotic detoxification. Modulation of signaling pathways associated with this transcription factor represents a major mechanism underlying tumor cell resistance to PDT [58,59]. PDT may also be accompanied by post-translational modifications affecting antioxidant system activity; for example, changes in SOD2 acetylation levels during aminolevulinic acid–induced PDT have been reported [60].

A common cause of resistance to various forms of drug therapy, including PDT, is the activity of membrane proteins responsible for xenobiotic efflux. Increased expression of ATP-binding cassette (ABC) transporters has been documented in many tumor types. The activity of these efflux pumps reduces intracellular accumulation of photosensitizers and, consequently, their efficacy. Tetrapyrrole photosensitizers are substrates for ABC transporters of the G subfamily, particularly ABCG2 [61,62,63,64]. This mechanism has been demonstrated for compounds of various structural classes, including porphyrins, benzoporphyrins, chlorins, and pheophorbide derivatives, as well as for aminolevulinic acid [65,66,67]. Evidence has also accumulated regarding the role of P-glycoprotein, a member of the ABC transporter B subfamily (also known as multidrug resistance protein 1, MDR1, or ABCB1), in the development of resistance to PDT [68,69]. Thus, in addition to the classical requirements for “ideal” photosensitizers, the need to minimize their transport by these carriers has been recognized. This may be achieved through chemical modification of photosensitizers or through the use of nanoscale delivery systems [66,70]. Importantly, these metabolic features may also be exploited for targeted therapeutic strategies. In particular, it has been proposed to utilize the ability of photosensitizers to bind ABCG2 and ABCB1, followed by irradiation, to achieve targeted inactivation of drug efflux transporters, thereby increasing tumor sensitivity to both PDT and other therapeutic modalities [71].

The endoplasmic reticulum (ER) plays an important role in cellular responses to PDT and in the development of resistance. The ER is highly sensitive to various stressors, including disturbances in energy metabolism, alterations in redox balance, and disruptions in Ca^2+^ homeostasis. ER dysfunction impairs protein folding, leading to the accumulation of misfolded proteins and the development of ER stress [72]. To counteract these disturbances, cells activate a set of protective mechanisms collectively referred to as the unfolded protein response (UPR). This signaling network is mediated by three transmembrane ER receptors: PERK (PKR-like ER kinase), ATF6 (activating transcription factor 6), and IRE1 (inositol-requiring enzyme 1). Under physiological conditions, these receptors remain inactive through association with the chaperone GRP78 (glucose-regulated protein, 78 kDa), which dissociates upon accumulation of misfolded proteins [73]. Activation of UPR signaling occurs sequentially. PERK is activated first and phosphorylates the translation initiation factor eIF2α, leading to global suppression of protein synthesis while enabling selective translation of activating transcription factor 4 (ATF4), which induces genes involved in restoration of ER function [74]. PERK also mediates inter-organelle communication by regulating ROS production and Ca^2+^ exchange between the ER and mitochondria [72]. The second ER stress sensor, ATF6, is activated through limited proteolysis following transport to the Golgi apparatus, and its active form regulates the expression of multiple chaperone proteins [75]. The third receptor, IRE1, possesses both serine–threonine kinase and endoribonuclease activities; its key functions include ER-associated mRNA degradation and processing of X-box binding protein 1 (XBP1) mRNA, which encodes another transcription factor involved in the UPR [76]. Depending on the severity of ER stress, activation of these pathways may result either in cell death or, less favorably in the therapeutic context, in the induction of autophagy, which promotes cell survival.

Increased resistance mediated by stress-response proteins, including chaperones and autophagy-related pathways, is associated with reduced PDT efficacy. To date, the involvement of several heat shock proteins (HSPs), including HSP27 [77], HSP70 [78], and HSP60 [79], in PDT resistance has been demonstrated. Recognition of the important role of autophagy in the cellular response to PDT has led to the development of new research directions focused on targeted modulation of autophagic pathways to reduce tumor cell survival. Numerous studies published in recent years highlight the significant potential of this approach [80,81,82,83].

Tumor cell survival during PDT and the development of resistance are associated with the capacity of DNA repair systems to maintain genome integrity and functionality. Many tumor types are characterized by increased activity of apurinic/apyrimidinic endonuclease 1 (APE1), which correlates with resistance to various antitumor agents. APE1 is a bifunctional enzyme involved, first, in DNA repair via the base excision repair (BER) pathway and, second, through its N-terminal domain, in the redox regulation of transcription factors. The primary form of DNA damage induced by PDT is base oxidation, which is predominantly repaired via the BER pathway [84]. Therefore, activation of this enzyme plays a major role in conferring cellular resistance [85,86]. To date, substantial clinical evidence has been accumulated demonstrating the potential of combining PDT with inhibitors of DNA repair proteins, including APE1, to overcome tumor cell resistance [87]. The redox activity of APE1 also contributes to resistance by regulating the activity of multiple transcription factors, including NF-κB, HIF-1α, AP-1, p53, STAT3 (Signal transducer and activator of transcription 3), NRF2 (Nuclear factor erythroid 2-related factor 2), and HSF1 (Heat shock factor 1) [88,89,90], thereby promoting cell growth, migration, survival, inflammation, and angiogenesis. p53 is a key protein responsible for maintaining genome integrity and regulating cell fate decisions, including proliferation, cell cycle arrest, and apoptosis. Up to half of malignant tumors harbor mutations in p53, enabling continued proliferation despite severe genetic damage and leading to genomic instability, tumor heterogeneity, and tumor progression [91]. Tumor cells deficient in p53 exhibit resistance to various therapeutic modalities, including PDT [92,93]. In addition to p53, the regulation of apoptosis depends on the balance between anti-apoptotic and pro-apoptotic proteins of the Bcl-2 (B cell lymphoma 2) family. Dysregulation of these proteins is also a common mechanism underlying resistance to PDT [94,95].

Thus, a new understanding of the causes of tumor resistance to PDT has emerged, replacing the previously dominant opposing view [63,86,95]. This shift has been driven by the elucidation of molecular mechanisms activated by PDT, recognition of their complexity and regulatory interactions, and partial clarification of how cells determine whether to initiate cell death. It has become evident that resistance to PDT is a multifactorial phenomenon, and its effective management requires a personalized approach, including detailed characterization of the molecular profile of the tumor undergoing PDT.

## 3. Mechanisms of Photoinduced Regulated Cell Death

Previously, it was believed that cell death induced by photodynamic exposure could proceed via three principal pathways: apoptosis, necrosis, and autophagy [2]. It is well established that the use of high concentrations of photosensitizers or high light doses leads to rapid, unregulated, and non-immunogenic necrotic cell death. In contrast, regulated cell death pathways—apoptosis and autophagy—were considered the most favorable forms of cell death [84].

**Figure 1:**
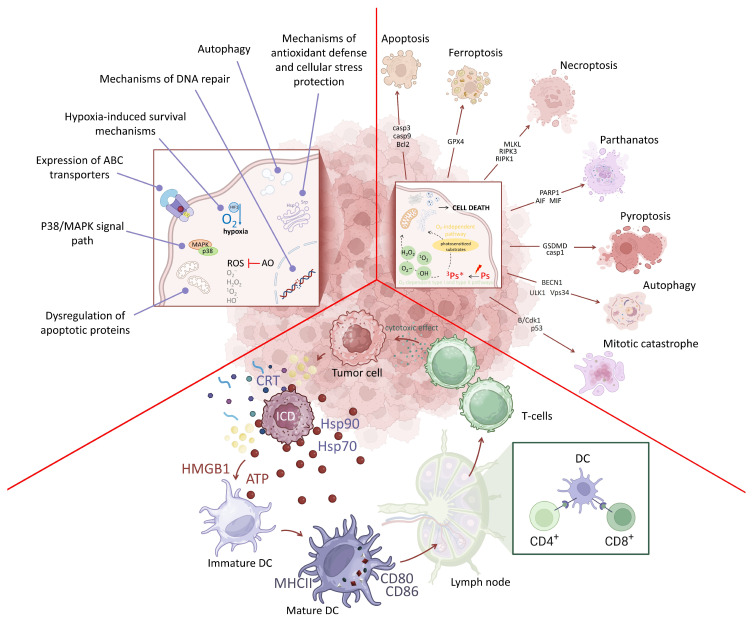
Diversity of outcomes of photodynamic treatment in tumor lesions. The tumor response to photodynamic therapy is determined by a combination of factors, including molecular genetic characteristics, degree of heterogeneity, lesion size, and other tumor features. Three fundamentally different outcomes of PDT in tumor tissue can be distinguished: (**upper left sector**) induction of therapy resistance, (**upper right sector**) activation of alternative cell death pathways, and (**lower sector**) development of immunogenic responses. Transitions between these scenarios are regulated by a complex set of determining factors. Experimental data indicate that the fate of malignant cells following PDT depends on their metabolic status, phenotypic characteristics, specific mutational landscape, and features of the microenvironment and secretory profile [103,104]. The key parameters determining the choice of cell death mechanism include the chemical nature of the photosensitizer, its intracellular localization, and irradiation parameters [103,104]. In particular, the induction of immunogenic cell death depends on the molecular profile of tumor cells, the biochemical properties and intracellular localization of the photosensitizer, and the intensity of exposure (i.e., the dose of the photosensitizer and the light dose). *, indicates an excited state.

Advances in methodological approaches to the study of cell death in recent years have significantly expanded our understanding of the molecular mechanisms underlying different cell death modalities and have led to the identification of new regulated forms of cell death. In 2018, the Nomenclature Committee on Cell Death (NCCD) described more than 10 modalities of regulated cell death (RCD) based on morphological features, molecular mechanisms, and immunological characteristics, and also referenced additional forms of cell death [96,97]. Since then, accumulating experimental data have expanded this initial list, and evidence has emerged for additional mechanisms, including mitoptosis [98], oxeiptosis [99], alkaliptosis [100], disulfideptosis [101], and cuproptosis [102]. An important conclusion of the NCCD was the recognition that regulated cell death can influence the microenvironment. This insight has opened new therapeutic perspectives in oncology, including the development of strategies aimed at modulating RCD modalities and designing agents capable of controlling immune responses for therapeutic purposes. Photodynamic therapy may act as such a “switch” between different cell death pathways (Figure 1).

The concept that PDT induces cell death exclusively through unregulated necrosis or regulated apoptosis was accompanied by a widespread assumption regarding the “advantages” of apoptosis induction [105]. Consequently, many studies have sought—and continue to seek—to demonstrate that the photodynamic effect induces apoptosis. However, whether apoptosis represents a universal and optimal mode of cell death remains an open question. In recent decades, it has been demonstrated that PDT-induced cell death can occur through alternative mechanisms. In particular, the induction of necroptosis, ferroptosis, pyroptosis, parthanatos, and other forms of cell death has been reported (Table 1). Notably, each of these modalities may exhibit either immunogenic or non-immunogenic properties [106].

Studies have demonstrated that the outcome of PDT in tumor cells is determined by their metabolic characteristics, phenotypic properties, mutational profile, and the influence of the microenvironment and secretome [95,128]. It appears that the nature of the photosensitizer, its concentration, and the mode of PDT exposure also influence the initiation of distinct cell death mechanisms [129,130]. Given the short lifetime and limited diffusion capacity of ROS, the effectiveness of PDT is largely determined by the intracellular localization of the PS, as this defines the primary targets of photodamage [131]. It has been established that ROS generation induced by endoplasmic reticulum (ER) stress is an important factor contributing to regulated cell death [38]. In addition, accumulation of PS in the Golgi apparatus has been shown to promote immunogenic tumor cell death [106].

The diversity of photoinduced cell death modalities has already reshaped the interpretation of classical PDT-based treatment protocols. Moreover, several studies have reported cell death exhibiting biomarkers of two distinct modalities simultaneously. This synergistic effect results in comparably pronounced outcomes [132,133,134].

### 3.1. Apoptosis

Apoptosis is a programmed form of cell death that occurs without disruption of the plasma membrane. Unlike necrosis, apoptosis prevents leakage of intracellular contents and, consequently, avoids the induction of inflammation in surrounding tissues.

Apoptotic mechanisms have been investigated using a wide range of first- and second-generation photosensitizers, including hypericin [107,135,136,137], Pc 4 [138], rose bengal [108], 5-aminolevulinic acid (5-ALA) [109,139,140], and benzoporphyrin derivatives (BPD-MA, verteporfin) [141].

For many years, apoptosis was considered the most desirable mechanism of programmed cell death in PDT due to the absence of adverse effects compared with necrosis. However, it is now known that tumor cells may exhibit resistance to apoptosis as a result of mutations in p53 and members of the Bcl-2 family.

### 3.2. Autophagy

Autophagy has been identified as an alternative mechanism of PDT-induced cell death and may become the predominant process under conditions of suppressed apoptotic pathways [142,143].

Sensitive targets for photosensitizers include anti-apoptotic proteins of the Bcl-2 family localized in mitochondria or the ER [144]. Reduced expression or functional inactivation of these proteins can initiate both apoptotic and autophagic cell death, as Bcl-2 normally inhibits key regulators of both processes (BAX/BAK in apoptosis and Beclin-1 in autophagy) [113].

Autophagy is generally a cytoprotective process that promotes cell survival. However, its role in tumor development is context-dependent and varies according to tumor stage, localization, type, and treatment conditions. Under different circumstances, autophagy may exert both tumor-promoting and antitumor effects. Thus, in the context of PDT, the role of autophagy depends on the PS used: in some cases, it enhances resistance by preventing cell death, whereas in others, it increases sensitivity by inducing autophagic cell death. Furthermore, activation of autophagy in tumor cells may reduce the efficacy of PDT by suppressing the antitumor immune response. Autophagy can be induced by Photofrin, m-THPC (meta Tetra(hydroxyphenyl)chlorin (Temoporfin)), verteporfin, AlPcS, and AlPcS2a (Aluminum phthalocyanine disulfonate, isomer a) [113,145,146,147].

### 3.3. Necroptosis

Necroptosis is a caspase-independent form of regulated cell death that phenotypically resembles necrosis. It is characterized by plasma membrane rupture, swelling of organelles and cytoplasm (oncosis), and release of intracellular contents, leading to inflammation [148].

The induction of necroptosis during PDT has been demonstrated using 5-aminolevulinic acid [149].

### 3.4. Paraptosis

Paraptosis is a caspase-independent form of cell death characterized by the formation of multiple vacuoles that progressively fill the cytoplasm. Morphologically, these processes are accompanied by extensive cytoplasmic vacuolization due to expansion of the ER and mitochondria and occur in the complete absence of caspase activation, which distinguishes paraptosis from apoptosis [117,150].

Paraptosis in tumor cells has been associated with verteporfin treatment and with chemophotodynamic therapy using m-TSX (meta- porphyrin based photosensitizer, TSX family) and p-TSX (para- porphyrin-based photosensitizer, TSX family) [116,151]. The potential significance of paraptosis as a mechanism for cancer control is supported by studies demonstrating that cell lines with impaired apoptotic pathways can be eliminated via paraptosis [152].

### 3.5. Ferroptosis

Ferroptosis is a form of programmed cell death dependent on intracellular iron levels, which catalyze the formation of ROS and subsequent oxidative damage to the cell [153,154].

Ferroptosis can be induced by PDT using Photosens, 5-ALA, and sodium talaporfin (TS-PDT) [118,119]. In addition, two cyclometalated Ir(III) complexes, IrL1 and MitoIrL2, reported in [119,120] as photosensitizers, are capable of inducing cell death via the ferroptotic pathway. Zhang et al. proposed the use of zinc oxide (ZnO) nanoparticles as carriers for the photosensitizer chlorin e6 [120,121]. It has been shown that ZnO nanoparticles can disrupt iron homeostasis and induce ferroptosis in tumor cells [121].

### 3.6. Parthanatos

Parthanatos is a caspase-independent form of programmed cell death caused by an excessive cellular response to DNA damage and mediated primarily by poly(ADP-ribose) polymerase 1 (PARP-1).

Morphologically, parthanatos is characterized by DNA fragmentation and nuclear condensation. At the subcellular level, cells undergoing parthanatos exhibit nuclear shrinkage and chromatin condensation [155].

Activation of PARP-1 can be triggered by reactive nitrogen species, such as NO, as well as ROS (particularly under photodynamic therapy conditions) [156]. Parthanatos has been shown to be induced by the photodynamic agent Na-H_2_TCPP in human breast cancer cells (MCF-10A) [111].

### 3.7. Pyroptosis

Pyroptosis exhibits features of both apoptosis and necrosis (without representing a hybrid form) and is characterized by formation of an inflammasome. The inflammasome is a multiprotein complex consisting of an adaptor protein (typically ASC), procaspase-1, and a sensor protein that responds to PAMPs and DAMPs (damage-associated molecular patterns), such as members of the NLR protein family. IL-1β and IL-18 are synthesized in cells as inactive precursors (pro-IL-1β and pro-IL-18) [157,158].

Pyroptosis can be induced by the IR700DX-6T photoprotector in colorectal cancer. Furthermore, membrane-targeted photoprotectors have been shown to induce pyroptosis [126,159].

### 3.8. Cuproptosis

Cuproptosis is a form of regulated cell death induced by excessive accumulation of copper (Cu) in mitochondria. Morphologically, cuproptosis is characterized by plasma membrane rupture, chromatin condensation or fragmentation, destruction of the endoplasmic reticulum, and mitochondrial shrinkage [102].

It should be noted that the Nomenclature Committee on Cell Death did not include cuproptosis as a distinct cell death modality in 2018. However, this form of cell death is now well established in numerous studies and is considered an independent alternative pathway [160,161,162].

Cuproptosis can be induced by a system based on CuO nanoparticles and the photosensitive compound TBP-2, coated with platelet membranes [163]. Xu et al. developed a GOx@[Cu(tz)] nanoplatform based on copper and triazole acid, which also effectively induces tumor cell death via the cuproptosis pathway [164].

Thus, tumor cells exposed to PDT may undergo multiple cell death pathways with either immunogenic or non-immunogenic properties. The therapeutic outcome of PDT critically depends on the specific cell death pathway activated and the associated immunogenic consequences.

## 4. Photoinduced Immunogenic Cell Death

Cell death during PDT under mild conditions may be accompanied by the active release of molecules capable of acting as adjuvants. One such cell death modality was first described by Garg et al. in 2012 [165]. It was already evident that the same photosensitizer, when combined with PDT, can lead to different cell death outcomes depending on its concentration and the applied light dose. Under relatively mild conditions, when cell death occurs through redistribution of photoactivated molecules within cellular compartments and gradual disruption of cellular membranes, cells initiate mechanisms of energy-dependent cell death [166]. As a result, ATP (Adenosine triphosphate), the nuclear protein HMGB1 (High mobility group box 1 protein), and ANXA (Annexin A protein family) undergo active transport. In addition to phosphatidylserine, a typical marker of regulated cell death, calreticulin and the heat shock proteins Hsp70 and Hsp90 are exposed on the cell surface. This form of cell death, accompanied by the release of DAMPs and activation of an adaptive immune response, is termed immunogenic cell death (ICD).

ICD is characterized not only by antigen presentation by dying cells but also by the release of endogenous adjuvant signals that promote the recruitment and activation of antigen-presenting cells. ICD can be induced by several cell death modalities, including apoptosis [106], necroptosis [167,168] and ferroptosis [169,170,171]. Other forms of cell death may also induce ICD; however, definitive evidence is still being accumulated [117,126,172].

Tumor infiltration by effector immune cells is also considered a marker of immunogenic cell death [173]. These infiltrating populations may include dendritic cells, CD8^+^ cytotoxic T lymphocytes, and natural killer (NK) cells. A key indicator of the immunogenicity of cell death is the ability of dying or dead cells to induce protective immunity against tumor challenge in vivo [172,174]. Immunogenic cell death represents a regulated form of cell death that culminates in activation of an antigen-specific adaptive immune response. This process leads to the establishment of immunological memory, which fundamentally distinguishes ICD from immunologically silent forms of cell death.

The type of cell death induced by PDT, whether immunogenic or non-immunogenic, depends on several factors, including the subcellular localization of the photosensitizer, the cell type and its physiological state, oxygen availability, and the PDT dose, defined by the combination of photosensitizer concentration and light dose.

The induction of an immune response following PDT was first described by Yamamoto et al. in 1991 [175]. They demonstrated that reactive oxygen species generated during photodynamic reactions induce lipid peroxidation in lymphocyte membranes, leading to macrophage activation via Fc receptors. An optimal PDT protocol should ensure effective destruction of the primary tumor through the induction of immunogenic cell death, thereby stimulating the immune system to recognize and eliminate residual tumor cells, including those at distant metastatic sites. Garg et al., in a 2016 study published in Science Translational Medicine, used hypericin to induce ICD in high-grade glioma in mice [176]. They showed that vaccination with ICD-treated tumor cells increased survival in tumor-bearing animals, with vaccination efficacy depending on DAMP production. Using Photosens, photoditazine, and tetra(aryl)tetracyanoporphyrazines, induction of immunogenic cell death was also demonstrated and was accompanied by the release of ATP and HMGB1. Furthermore, incubation of dying cells with antigen-presenting cells derived from mouse bone marrow resulted in their activation [106,177,178].

In recent years, accumulating evidence has indicated that PDT is a promising approach for inducing immunogenic cell death in experimental oncology. However, most studies have been conducted in mouse models, and validation of these findings in clinical trials is now essential. Nevertheless, investigation of the fundamental mechanisms underlying the immunogenicity of cell death remains an important task. Apoptosis, traditionally regarded as a non-inflammatory form of cell death, generally exhibits weak immunogenicity due to rapid clearance of apoptotic cells by phagocytes and minimal DAMP release [179]. However, in oncology, apoptosis, despite its role as a target of photodynamic therapy, is limited in its capacity to induce immunogenic cell death. Therefore, preclinical and clinical studies are actively investigating the combination of apoptosis-inducing modalities with activators of more immunogenic pathways, such as ferroptosis, pyroptosis, and necroptosis. The combination of these forms of cell death enhances the efficacy of antitumor immunotherapy by increasing oxidative stress, activating CD8^+^ T cells, and promoting DAMP release, thereby contributing to a reduced risk of recurrence [179]. Autophagy, in contrast, plays a context-dependent role: on the one hand, it can support tumor survival and suppress the immune response [180,181]; on the other hand, it can promote cell death (autophagic cell death) and enhance sensitivity to other forms of regulated cell death, acting as an activator [182].

Among the forms of cell death, pyroptosis, necroptosis, ferroptosis, and the more recently identified parthanatos and cuproptosis are characterized by high immunogenicity. Pyroptosis and necroptosis are accompanied by disruption of membrane integrity and the release of DAMPs, including ATP, HMGB1, DNA, and phosphatidylserine. This enhances dendritic cell maturation, production of pro-inflammatory cytokines, antigen cross-presentation, and formation of antitumor CD4^+^/CD8^+^ T-cell clones, thereby increasing the efficacy of therapies, including PD-1/PD-L1 inhibitors combined with chemotherapy or radiotherapy [183,184]. Ferroptosis, associated with the accumulation of peroxidized lipids, is accompanied by calreticulin exposure and HMGB1 release, which enhance tumor cell uptake by dendritic cells, activate STING/IFN-γ-dependent pathways, CD8^+^ T cells, and NK cells, and enable the use of ferroptotic lysate as a source of antigens for dendritic cell vaccination [185]. Parthanatos, induced by PARP-1 activation and NAD^+^/ATP deficiency, and cuproptosis, induced by copper excess, are also accompanied by the generation of poly(ADP-ribose) (PAR), mitochondrial DNA, ROS, and protein aggregates, as well as the release of HMGB1 and other DAMPs. This promotes dendritic cell maturation, CD8^+^ T-cell infiltration, and enhancement of systemic antitumor immune responses, although the immunogenic profile of parthanatos has not yet been fully characterized [155,186,187,188,189,190].

PDT strategies that activate pyroptosis, cuproptosis, and ferroptosis are the most promising for enabling in situ vaccination and enhancing immunotherapy in clinical practice. Apoptosis and autophagy are generally inferior to other immunogenic forms of regulated cell death [191,192].

It should be noted that under hypoxic conditions, the efficacy of PDT in inducing immunogenic cell death is reduced. Therefore, optimization of PDT efficacy under limited oxygen availability is of considerable importance [193]. Moreover, PDT itself contributes to the exacerbation of hypoxia within the tumor microenvironment (TME), thereby promoting angiogenesis, tumor progression, and metastasis, as well as reducing therapeutic efficacy and worsening prognosis [194]. More detailed investigation of PDT in combination with oxygen-enhancing strategies may provide new opportunities for the development of innovative approaches in cancer immunotherapy [195].

Several strategies have been proposed to overcome resistance in hypoxic tumors. One approach involves the development of an oxygen-enhanced nanocarrier containing chlorin e6 (C@HPOC), which is self-sufficient in oxygen supply [196]. This modified PDT strategy with enhanced oxygen availability demonstrated increased infiltration of cytotoxic T lymphocytes (CTLs) into the TME and more effective induction of immunogenic cell death in 4T1 breast cancer cells in mice. This effect was associated with increased surface exposure of calreticulin (CRT), enhanced release of HMGB1 and ATP, and subsequent activation of dendritic cells. Alternative approaches utilize perfluorocarbon-based nanoparticles, or nanodroplets, in so-called Oxy-PDT. Due to their high oxygen-carrying capacity, perfluorocarbons can maintain higher oxygen levels than the tumor matrix at a given partial pressure. This strategy has demonstrated greater therapeutic efficacy of Oxy-PDT compared with conventional PDT, both in vitro and in vivo [193].

Activation of the immune system by PDT has been the focus of intensive research in recent years. This has not only contributed to a deeper fundamental understanding of the underlying mechanisms in experimental studies but has also laid the foundation for clinical applications.

## 5. Modification of Dendritic Cell Antitumor Vaccines with Photoinduced Cells

The induction of immunogenic cell death during photodynamic therapy is achievable in vivo. In this context, tumor cells undergoing ICD function as an endogenous vaccine, stimulating adaptive immunity. However, this approach has a limited scope of application and can be used only for neoplasms amenable to PDT. Such tumors include skin cancers, gynecological tumors (including cervical cancer), and malignancies of the esophagus, lung, bladder, gastrointestinal tract, and oral cavity. The group of Patrizia Agostinis was among the first to propose loading dendritic cells with tumor cells undergoing immunogenic cell death following photodynamic therapy and to apply this approach as immunotherapy for glioma [176]. In 2016, they demonstrated that dendritic cell vaccines could represent a successful strategy for harnessing the properties of photodynamically induced immunogenic cell death.

Dendritic cells are capable of inducing an adaptive antitumor immune response through the uptake and presentation of tumor-specific antigens (TSAs), migration to lymph nodes followed by priming of T lymphocytes, and recruitment and activation of tumor-infiltrating T cells. This was demonstrated in 1995 in both in vitro [197] and in vivo studies [198,199]. The production of modern dendritic cell-based therapeutics is based on the generation of dendritic cells from peripheral blood monocytes of the patient under in vitro conditions. These cells are subsequently cultured with a combination of growth factors and loaded (co-cultured) with tumor antigens, including patient-derived tumor proteins, synthetic peptides, or tumor-derived genetic material. The cells are then induced to mature in order to enhance their capacity to activate the immune system. Finally, the resulting vaccine is administered to the patient, typically via subcutaneous or intradermal injection, after which dendritic cells migrate to lymph nodes and prime T lymphocytes to recognize and eliminate target cells.

Various forms of tumor antigen delivery to dendritic cells (DCs) are used, including lysates of autologous tumor cells, isolated tumor-associated peptides/proteins, or synthesized DNA/RNA constructs and peptides/proteins. Autologous tumor cells contain a complete repertoire of individual mutant TSAs, including those potentially critical for the antitumor response. In contrast, tumor-associated antigens (TAAs) are predominantly expressed in tumor cells but are also present at low levels in certain normal tissues. Unlike TSAs, TAAs are not unique to cancer; rather, they represent either overexpressed normal proteins or proteins associated with embryonic development. Therefore, the use of individualized stimulatory proteins or mRNA encoding patient-specific TSAs for vaccine loading is of particular importance, although this issue remains subject to debate [200]. TSAs are often referred to as tumor neoantigens. Tumor neoantigens are proteins generated as a result of mutations in tumor cells that can be processed and presented by antigen-presenting cells, thereby eliciting a specific immune response [201]. T cells induced by neoantigens, rather than TAAs, do not contribute to the development of autoimmune reactions. Thus, neoantigens represent a critical component of immune surveillance in tumor tissue during the induction of immunogenic cell death; however, they are not the sole contributing factor.

The use of tumor lysates, typically derived from heat shock–treated cells, as a source of tumor neoantigens has a potential advantage, as this approach can induce a polyclonal immune response by stimulating both CD4^+^ and CD8^+^ T cells [202,203,204]. However, despite the theoretical efficacy of such autologous vaccines, their clinical effectiveness has proven to be lower than expected. In 2010, the only dendritic cell vaccine demonstrating sufficient efficacy against prostate cancer was approved [205]. Nevertheless, the primary limitation of this vaccine remains the modest extension of patient survival.

The development of improved DC-based therapeutic strategies may enhance therapeutic efficacy while maintaining a favorable safety profile. Several research groups worldwide have demonstrated the efficacy of dendritic cell vaccines developed based on the concept of immunogenic cell death induced by photodynamic therapy in various tumor models [176,206,207,208]. It has been shown that tumor cells subjected to PDT release danger-associated signals, including ATP, CRT, HSP70, HSP90, and HMGB1 [207,209,210]. In addition, dendritic cell maturation into an effector phenotype, characterized by the expression of CD80, CD86, CD40, and MHC II molecules in vitro as well as the presence of antitumor cytokines such as IL-12 and TNF-α (but not IL-10) in serum following administration, has been convincingly demonstrated [211]. In a comparative analysis of dendritic cell vaccines based on ICD versus DCs loaded with autologous lysate, a significant increase in immunogenicity and antitumor response was observed for ICD-induced vaccines in experimental tumor models [207,209,212].

According to accumulated clinical data, many therapeutic effects in patients with solid tumors are not fully realized due to the inhibitory influence of the immune microenvironment on dendritic cell proliferation and differentiation. Therefore, administration of activated dendritic cells, matured in vitro and capable of activating suppressed T cells, helps compensate for tumor-induced immunosuppression and enhances the adaptive antitumor immune response in patients. However, although some studies have demonstrated that dendritic cell vaccines can contribute to improvement of the tumor microenvironment [174,213,214,215], these effects are not consistently associated with improved clinical outcomes.

**Figure 2:**
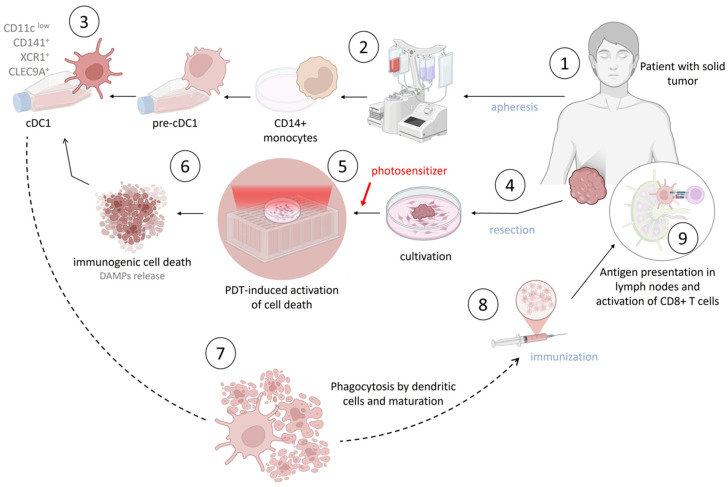
Schematic representation of the generation and mechanism of action of a dendritic cell vaccine against solid tumors. (**1**) Apheresis is used to obtain a monocytic fraction (CD14^+^ monocytes) from the patient’s peripheral blood, (**2**) followed by in vitro stimulation, (**3**) resulting in the generation of effector dendritic cell populations (CD11c^low^+^ CD141^+^ XCR1^+^ CLEC9A^+^ DC1). (**4**) Surgical tumor resection is followed by in vitro maintenance of tissue or cell culture viability. (**5**) Tumor cells are loaded with a photosensitizer, and photoactivation of cell death is induced by irradiation at an appropriate wavelength. (**6**) Photoinduced cell death may exhibit immunogenic properties and be accompanied by the release of damage-associated molecular patterns (DAMPs), which stimulate adaptive antitumor immune responses. (**7**) Co-cultivation of dying tumor cells with isolated dendritic cells results in phagocytosis of cellular debris. (**8**) Consequently, dendritic cells present tumor antigens via MHC II complexes to T lymphocytes in lymph nodes. (**9**) This results in the establishment of immunological memory and differentiation of T lymphocytes into effector cytotoxic CD8^+^ T cells.

The proposed approach assumes that dendritic cell vaccines can be generated via apheresis of peripheral blood obtained from a patient or a compatible healthy donor. The resulting mononuclear cells are stimulated in vitro to differentiate into mature dendritic cells capable of antigen presentation. Resected tumor material is then subjected to in vitro photodynamic therapy, and immunogenic dying cells are phagocytosed by antigen-presenting cells during co-cultivation. Upon subcutaneous administration, the vaccine migrates to regional lymph nodes and stimulates T-cell recruitment and the formation of immunological synapses. This leads to activation of antigen-specific adaptive antitumor immunity and suppression of tumor cells by CD8^+^ T lymphocytes migrating to the tumor site (Figure 2).

To date, three key aspects remain insufficiently investigated: the optimal dendritic cell subset for vaccine development, the effects of dendritic cell vaccination on the tumor microenvironment, and the appropriate regimen for clinical application [216,217]. Some studies have demonstrated a transition from monocyte-derived DCs, which exhibit functional limitations in vitro, to alternative subsets such as cDC1, which display an enhanced capacity to activate effector cytotoxic CD8^+^ T cells [218,219]. The selection of suboptimal DC subsets, as well as the use of insufficiently immunogenic tumor antigens, may have contributed to the limited efficacy of previously developed vaccines. Furthermore, investigation of strategies to overcome the immunosuppressive tumor microenvironment may help to fully realize the therapeutic potential of DC-based vaccines [217,220].

The combination of the aforementioned approaches, together with promising results obtained in pilot preclinical studies, supports the continued development of novel immunotherapeutic strategies. This may facilitate the integration of PDT-based approaches into clinical practice while maintaining the principles of personalized medicine.

## 6. Dendritic Cell Vaccines as an Approach to Overcoming the Limitations of PDT

Despite significant progress in the development of experimental photodynamic therapy approaches and promising preclinical findings, the widespread implementation of these methods in routine clinical practice remains an unresolved challenge. The application of PDT is constrained by a complex set of interrelated factors, including the limited bioavailability of photosensitizers (their tendency to aggregate and low solubility), insufficient selectivity of delivery to tumor targets, associated systemic phototoxicity, as well as physical and physiological limitations such as restricted light penetration and the hypoxic tumor microenvironment [221].

In contrast to PDT, tumor therapy based on dendritic cell vaccines does not depend on the local physicochemical effects of light and photosensitizers within tumor tissues, but instead relies on the immune system as the primary effector mechanism. Moreover, the use of DC vaccines enables separation of the photodynamic stage from the stage of antitumor effect realization, thereby significantly reducing dependence on irradiation parameters and photosensitizer distribution within the tumor. As PDT is primarily employed in an ex vivo format to generate immunogenic tumor material, its role in this context is that of an auxiliary tool for the induction of immunogenic cell death rather than the sole method of direct tumor destruction in vivo [207,209,222,223].

### 6.1. Approaches to Overcoming the Problems of Photosensitizer Solubility and Aggregation

The primary limitations of PDT are associated with the physicochemical properties of photosensitizers, including low solubility in aqueous media, poor permeability and insufficient accumulation in tumor cells, and a pronounced tendency to aggregate, which impairs their function [221,224]. It is well established that many classical porphyrin- and chlorin-based photosensitizers are highly hydrophobic and prone to self-aggregation and require specialized carriers to improve pharmacokinetics and tumor distribution. These physicochemical limitations directly reduce the quantum yield of singlet oxygen and other reactive oxygen species, leading to heterogeneity of the cytotoxic effect both within individual tumors and between patients [225,226].

In the context of dendritic cell-based vaccine development, limitations related to photosensitizer solubility and aggregation can be mitigated by employing PDT in an ex vivo format for tumor cell treatment, where such constraints can be addressed through controlled experimental conditions. Since the photosensitizer solution is used only during vaccine preparation and the dendritic cells themselves are administered to the patient, limitations associated with photosensitizer solubility can be overcome by using solvents of different chemical natures at concentrations appropriate for experimental purposes. For example, under ex vivo conditions, organic solvents (such as polyethylene glycol, PEG), surfactants (including polysorbates and PEG-based surfactants), and co-solvents (e.g., ethanol) can be selected at concentrations that are non-toxic to tumor cell cultures and dendritic cells yet sufficient to maintain the photosensitizer in a monodisperse state within the culture medium. In addition, cyclodextrins are employed to enhance the solubility and photostability of photosensitizers in aqueous environments by forming inclusion complexes with hydrophobic molecules. For certain photosensitizers (e.g., hypericin), incorporation into β-cyclodextrin polymers has been shown to increase aqueous solubility, reduce aggregation, and provide more reproducible photodynamic activity. Such complexes are suitable for short-term in vitro treatment of tumor cells prior to the collection of lysates for dendritic cell loading. Thus, in the development of dendritic cell vaccines, a broader range of organic solvents, surfactants, and related agents can be employed to improve solubility and control the aggregation state of photosensitizers, since the final product is a dendritic cell vaccine that does not contain problematic amounts of photosensitizer in the patient’s body and does not induce the systemic phototoxicity characteristic of conventional PDT [12,223,227,228,229].

Experimental models of cutaneous squamous cell carcinoma have demonstrated that lysates of tumor cells killed by ALA-PDT can be effectively used for dendritic cell loading. The toxicity of solvents and the photosensitizer itself is not observed in vivo, as only antigen-loaded dendritic cells are administered during vaccination. This approach allows the use of higher local concentrations of photosensitizers and optimal solvent systems during the generation of immunogenic material, without transferring the associated risks to the patient [212,229].

### 6.2. Bypassing Limitations in Photosensitizer Delivery to Tumor Cells

Limited permeability of photosensitizers into tumor cells and their heterogeneous accumulation within tumor tissue are recognized as key determinants of the clinical efficacy of photodynamic therapy. Numerous studies have demonstrated that low selectivity and insufficient intracellular concentrations of photosensitizers result in a substantial proportion of tumor cells receiving only minimal amounts of reactive oxygen species due to inefficient generation, whereas normal tissues may accumulate significantly higher levels of the drug [226,230,231]. These limitations arise from several factors, primarily related to the intrinsic properties of photosensitizers. Many clinically used photosensitizers, particularly tetrapyrrole-based compounds, are highly hydrophobic, prone to self-aggregation, and poorly soluble in biological fluids; consequently, they exhibit limited distribution in the aqueous phase and insufficient penetration across the plasma membrane at effective concentrations [12,223,227,228,229]. Another important limiting factor is the vascular architecture of tumor tissue, which is often characterized by impaired vascularization and reduced blood flow, thereby restricting photosensitizer delivery to certain tumor regions [230,232]. In addition, the tumor microenvironment is highly heterogeneous: a dense extracellular matrix, increased cellular density, hypoxia, and tissue acidity all impede the penetration of photosensitizers into deeper tumor regions. As a result, even at identical systemic doses, different areas within the same tumor accumulate the drug unevenly, leading to regions with reduced PDT efficacy. Collectively, these factors result in a situation in which, despite appropriate systemic dosing, a significant fraction of tumor cells receives insufficient intracellular concentrations of photosensitizers in clinical settings [226,230,232,233].

When PDT is applied not as a standalone therapy but as a tool for generating material for dendritic cell vaccines, limitations associated with poor in vivo permeability and accumulation of photosensitizers can be circumvented by optimizing all stages of cell processing in vitro and shifting the primary antitumor effect to the immune response. For example, in vitro conditions allow precise control over parameters of photosensitizer incubation with tumor cells, including photosensitizer concentration, solvent composition and combinations, incubation time, and irradiation conditions [212,229]. It has been demonstrated that optimization of PDT parameters (photosensitizer dose, light power density, and incubation time) enables induction of immunogenic tumor cell death, accompanied by enhanced release of DAMPs and more effective stimulation of dendritic cells compared with necrotic lysates [225,229].

Importantly, dendritic cell vaccines eliminate the requirement for efficient photosensitizer accumulation within the patient’s tumor in vivo, as PDT is performed ex vivo on tumor cells or lysates, after which antigen-loaded dendritic cells, rather than the photosensitizer itself, are administered. This approach also prevents photosensitizer accumulation in normal tissues. Thus, dendritic cell-based vaccination shifts the key requirement from “maximal photosensitizer accumulation within the patient’s tumor” to “controlled photosensitizer accumulation and high-quality induction of immunogenic cell death in vitro,” which is more readily standardized and optimized experimentally.

### 6.3. Reducing Systemic Phototoxicity by Transferring PDT to an Ex Vivo Format

In conventional photodynamic therapy, a photosensitizer is administered systemically and circulates throughout the body, accumulating in both tumor and normal tissues. Upon exposure to light, this may result in phototoxic reactions that can persist for several weeks. This significantly limits the application of PDT in vivo due to phototoxicity and potential thermal damage. Clinical reviews indicate that many systemically administered photosensitizers cause prolonged cutaneous and ocular photosensitivity, requiring patients to strictly avoid sunlight and intense artificial light for extended periods, thereby reducing the safety and tolerability of the therapy. Furthermore, insufficient selectivity of photosensitizer accumulation in tumor tissue leads to damage of surrounding normal tissues, photothrombotic reactions in blood vessels, and increased local inflammation, particularly when higher light doses are applied to compensate for insufficient therapeutic efficacy. To mitigate these adverse effects, targeted photodynamic systems (e.g., conjugates with antibodies or ligands targeting tumor receptors) and nanocarriers (liposomes, micelles, polymeric nanoparticles) are being actively developed. However, even with these approaches, the risk of residual systemic phototoxicity is not completely eliminated [221,224].

The clinical use of dendritic cell vaccines is not associated with these limitations, as neither systemic administration of photosensitizers nor in vivo photodynamic activation is required. Moreover, dendritic cell vaccination does not necessitate the use of high-intensity irradiation at specific tissue depths; it is sufficient to apply conditions that induce immunogenic cell death ex vivo during vaccine preparation. As a result, the photodynamic component in DC vaccines functions as a tool for generating antigenic material, whereas the actual antitumor effect is mediated by systemic T-cell and auxiliary immune responses, without additional phototoxicity to normal tissues. This confers a significantly more favorable clinical safety profile compared with conventional PDT, in which the need to control photosensitivity and the risk of skin and ocular damage substantially limit treatment regimens and frequency [221,224].

### 6.4. Circumventing the Limited Penetration Depth of Light

The limited penetration depth of visible and near-infrared light into biological tissues remains one of the fundamental and unresolved technical barriers to the widespread clinical application of PDT, particularly in the treatment of deep-seated and large solid tumors. In most soft tissues, the intensity of red light (approximately 630–700 nm), commonly used to activate clinical photosensitizers, decreases exponentially, resulting in an effective penetration depth of only 1–3 mm. From a clinical perspective, this implies that PDT is primarily effective for superficial lesions (e.g., skin, mucous membranes, and accessible hollow organs), whereas deeper tumor regions, large tumor masses, and metastases located beyond the effective irradiation zone remain inaccessible for adequate photosensitizer activation. This limitation leads to heterogeneous treatment outcomes: peripheral tumor regions receive sufficient light exposure and are damaged, whereas central hypoxic and poorly accessible areas often remain viable and may serve as sources of tumor recurrence and progression [234,235,236,237].

Consequently, limited light penetration is regarded as a major obstacle to the development of PDT as a universal therapeutic modality for internal and metastatic tumors, driving the exploration of alternative strategies such as internal light sources, radiation- or chemiluminescence-based activation systems, and combined immunotherapeutic approaches [223,226,234,238].

When PDT is applied as a tool for generating immunogenic tumor material in the development of dendritic cell vaccines, this limitation can be effectively circumvented by performing photodynamic treatment in vitro under controlled cell culture conditions. This enables the induction of immunogenic cell death and the generation of DAMP- and antigen-rich material for dendritic cell loading. Subsequently, the resulting dendritic cell vaccine can exert antitumor effects against primary tumors and metastases regardless of their depth or accessibility to light [212,223,229].

### 6.5. Shifting the Focus from Oxygen-Dependent Cytotoxicity to Tumor Immune Control

Tumor hypoxia remains a key and fundamental limitation of the effectiveness of clinical photodynamic therapy, as most photosensitizers exert their cytotoxic effects via oxygen-dependent mechanisms (type II), based on the generation of singlet oxygen and other reactive oxygen species. In many solid tumors, baseline oxygen levels are already reduced due to impaired vascularization, increased oxygen consumption by rapidly proliferating cells, and structural features of the tumor microenvironment. During PDT, this condition is further exacerbated by additional oxygen consumption and microvascular occlusion, which further limit ROS generation. Clinically, this results in reduced sensitivity of hypoxic tumors to PDT, heterogeneous responses within a single tumor lesion, and an increased likelihood of survival of tumor cell subpopulations adapted to low-oxygen conditions, which are associated with disease recurrence and progression. For this reason, tumor hypoxia is regarded as a major limitation of PDT and represents a central focus of current research [239,240,241].

When PDT is used as an initiator of immunogenic cell death in vitro in the context of dendritic cell-based vaccines, it induces tumor cell damage and promotes the release of various DAMPs (CRT, HMGB1, ATP, HSP), which are subsequently captured by dendritic cells during co-cultivation. Because these procedures are performed in vitro using tumor cell cultures, oxygen conditions can be more favorably controlled, including adequate medium aeration and regulation of cell layer thickness [223,225]. In addition, strategies may be employed to increase oxygen availability in the culture medium, such as the use of oxygen carriers or donors (e.g., perfluorocarbons or catalytic nanoparticles that decompose H_2_O_2_ to O_2_), thereby enhancing ROS generation during PDT and improving the quality of immunogenic cell death [48,242]. Alternatively, photosensitizers capable of inducing type I photochemical reactions (radical-based mechanisms) may be used [243].

Thus, in the context of PDT applied for the preparation of tumor lysates for dendritic cell vaccines, oxygen is required only to generate high-quality immunogenic tumor material under controlled conditions. Following administration of the prepared dendritic cell vaccine, the primary antitumor effect in vivo is mediated by cytotoxic T-cell responses and other immune effector mechanisms that are not dependent on the presence of molecular oxygen within the tumor. Moreover, such vaccines have been reported to reduce the risk of tumor recurrence and to control distant metastases, where local light exposure and efficient singlet oxygen generation are technically infeasible [103,104,244,245].

### 6.6. Transforming the Local Effect of PDT into a Systemic Immune Response Using DC Vaccines

The absence or insufficiency of an effective antitumor immune response following photodynamic therapy in clinical settings represents a significant limitation, as the immune component is critical for long-term tumor control, prevention of relapse, and elimination of micrometastases beyond the irradiation field. Although PDT has been shown experimentally to induce immunogenic cell death and the release of DAMPs (CRT, HMGB1, ATP), as well as to activate both innate and adaptive immunity, in practice this response is often transient, partially suppressed by regulatory T cells and other immunosuppressive components of the tumor microenvironment, and does not translate into sustained T cell–mediated disease control. Clinical and preclinical studies indicate that, in the absence of additional immunomodulation, PDT rarely induces a robust systemic T-cell response or the formation of durable antitumor immune memory. Its effects are predominantly limited to local tumor destruction within the treatment field, whereas distant lesions and minimal residual disease remain sources of progression. Moreover, PDT may induce not only immune activation but also a compensatory expansion of immunosuppressive regulatory T-cell populations, further limiting endogenous antitumor immunity and driving the development of combination therapeutic strategies to overcome this barrier [222,246,247].

In the context of dendritic cell-based vaccines, the limitations of conventional PDT—namely, spontaneous and insufficiently controlled immune activation within the tumor—are replaced by a controlled and optimized process of antigen presentation mediated by professional antigen-presenting cells. In DC vaccine therapy, antigen uptake and presentation are achieved through optimized in vitro culture of dendritic cells using lysates derived from PDT-treated tumor cells, which promotes immune cell activation and maturation. To further enhance dendritic cell functionality, adjuvant agents are employed to increase the expression of costimulatory molecules on the cell surface (e.g., CD80, CD86). Following administration, dendritic cells migrate to lymph nodes, where they actively interact with other immune cells, thereby amplifying the antitumor immune response. In particular, dendritic cell vaccines based on PDT-treated tumor cells have been shown to enhance infiltration of CD8^+^ T lymphocytes into tumors, increase IFN-γ production, and exhibit synergistic effects with immune checkpoint antibody therapy, such as anti-PD-L1 [225,245].

Thus, the therapeutic efficacy of dendritic cell vaccines does not depend on the physicochemical constraints of photodynamic therapy (e.g., oxygen availability, light penetration, photosensitizer solubility) but instead transforms PDT into a tool for the controlled generation of immunogenic tumor material for subsequent systemic antitumor immunization. Experimental evidence from models of cutaneous squamous cell carcinoma, glioblastoma, and head and neck squamous cell carcinoma demonstrates that this combined approach results in more pronounced tumor growth inhibition and improved survival compared with either PDT alone or conventional dendritic cell vaccines lacking immunogenic cell death induction [176,229,245]. Furthermore, this therapeutic strategy enables rational combinations of dendritic cell vaccines with other immunotherapeutic modalities (including checkpoint inhibitors (ICIs), chemotherapy, and radiotherapy) aimed at enhancing systemic T-cell responses and controlling minimal residual disease. In the long term, the “PDT + DC vaccine” approach represents a promising platform for personalized treatment of patients with solid tumors, particularly in cases where the physical and biological limitations of conventional PDT preclude a sustained clinical response [212,245,248].

## 7. Concluding Remarks

Until recently, efforts to improve the efficacy of photodynamic therapy were largely focused on the search for an “ideal” photosensitizer. However, it is becoming increasingly evident that such a photosensitizer is unlikely to be identified. Consequently, recent research has shifted toward the modification of existing PDT protocols and the development of new strategies. Given that PDT is a highly effective approach for eliminating tumor cells, current efforts are aimed at harnessing its beneficial properties while identifying ways to overcome the challenges associated with its application. The advantageous features of photoinduced cell death are largely attributable to the ability of PDT to activate multiple mechanisms of cell death, bypass resistance to chemotherapy, and, importantly, induce immunogenic cell death. Notably, PDT can activate apoptotic pathways even in cells that are resistant to apoptosis induced by chemotherapy or radiotherapy [249].

If an ideal photosensitizer does not exist, then the way forward must be more engineering-oriented than molecular. This is precisely why contemporary research efforts have shifted toward the development of nanocarriers: not to replace or abandon the photosensitizer but to overcome the limitations of photodynamic therapy—poor solubility, oxygen dependence, and shallow light penetration—through advanced delivery platforms.

The use of nanocarriers (nanoparticles, liposomes, hydrogels, dendrimers, micelles, and other platforms) enables these limitations to be addressed by improving solubility, enhancing biocompatibility, and providing controlled delivery of photosensitizers to target tissues. Liposomes are biocompatible phospholipid nanovesicles capable of encapsulating both hydrophilic and hydrophobic photosensitizers and accumulating passively in tumors [250]. Activation of the photosensitizer by near-infrared (NIR) light leads to ROS generation and subsequent liposome disruption (or enzymatic release), enabling localized drug delivery while minimizing systemic toxicity [251]. However, key limitations include low photosensitizer loading capacity and instability in the bloodstream (premature leakage). Polymeric nanoparticles improve biodistribution and permeability due to their controllable size and surface chemistry, enabling targeted delivery while protecting the photosensitizer from premature degradation [252,253]. Their main limitation is the technological complexity of large-scale production. Dendrimers are nanoscale branched polymers with a monodisperse structure and tunable surface, capable of encapsulating photosensitizers within their core or conjugating them on the surface [254,255]. Due to their high loading capacity and stability, they improve tissue penetration and reduce systemic toxicity (e.g., for 5-ALA delivery). However, their limitations include multistep synthesis, high cost, and uncertain biocompatibility.

Inorganic nanocarriers are also widely studied. Gold nanoparticles (AuNPs) represent a versatile platform, synthesized in various forms (spheres, rods, shells) with strong absorption in the near-infrared range, allowing their use in both PDT and photothermal therapy. Their surfaces can be readily functionalized with ligands and photosensitizers for both passive and active delivery [256,257,258]. The main limitation is slow clearance from the body, which increases the risk of chronic toxicity. Silica nanoparticles, particularly mesoporous ones (MSNs), are valued for their non-toxicity, optical transparency, and ease of chemical functionalization via surface hydroxyl groups [259,260]. Their high loading capacity and tunable porosity enable stable encapsulation and controlled release of photosensitizers; however, their non-biodegradability raises concerns regarding long-term accumulation. Quantum dots are zero-dimensional nanomaterials that, due to quantum confinement effects, exhibit broad absorption spectra, narrow emission ranges, and the ability to activate photosensitizers via FRET or upconversion, enabling precise tumor imaging and effective PDT in the near-infrared range [261]. However, their clinical application is limited by heavy metal toxicity (cadmium), low photosensitizer loading capacity, and poor solubility in aqueous environments [262,263].

Nanocarriers have achieved substantial progress in photodynamic therapy. However, all such systems face fundamental limitations. First, PDT remains oxygen-dependent, while hypoxia is frequently present in the core of solid tumors, reducing therapeutic efficacy. Second, light penetration into tissues is limited. Third, nanoparticles are rapidly taken up by the reticuloendothelial system, with up to 90% of the administered dose accumulating in the liver and spleen. Fourth, systemic phototoxicity remains a concern, requiring patients to avoid bright light for extended periods. In addition, each carrier type has specific limitations: liposomes have low loading capacity; polymeric particles are difficult to scale up; quantum dots contain toxic cadmium; and gold and silica nanoparticles exhibit slow clearance. Nevertheless, nanocarriers provide a pathway toward overcoming the most significant clinical limitation of PDT—its localized nature—through combination with immunotherapy. PDT performed using nanoparticles induces immunogenic cell death; however, in practice, this immune activation is suppressed by regulatory mechanisms (PD-1/PD-L1, regulatory T cells). Co-delivery strategies within a single nanoparticle offer a solution—for example, liposomes can simultaneously carry a photosensitizer and an immune checkpoint inhibitor [264,265,266]. Such hybrid systems have demonstrated in mouse models that PDT effectively destroys the primary tumor, while the checkpoint inhibitor relieves T-cell suppression, enabling systemic antitumor responses against distant metastases—an effect not achievable by either approach alone. Even more promising are strategies involving oxygen-generating nanoparticles (catalase or MnO_2_), which simultaneously address tumor hypoxia and enhance ICD [267,268]. Alternatively, nanoplatforms may be combined with oxygen carriers, which can also promote active ICD processes [38,196]. Thus, future research is likely to focus not on the development of additional nanoparticle systems, but rather on integrated therapeutic platforms that combine PDT, hypoxia modulation, and localized delivery of immunomodulators, thereby transforming a local photodynamic effect into a systemic anticancer immune response.

Among immunophotodynamic strategies, co-delivery systems combining photosensitizers with immune adjuvants are particularly noteworthy. These systems aim to induce tumor ICD and activate adaptive immunity against tumor-associated antigens [269,270]. However, they differ from dendritic cell vaccination in the location, timing, and mechanism of antigen presentation. Dendritic cell vaccines involve ex vivo loading of dendritic cells with antigens derived from PDT-treated tumor cells, providing a complete repertoire of patient-specific antigens and enabling antigen presentation independent of the suppressive tumor microenvironment. In contrast, co-delivery systems introduce the photosensitizer and adjuvant directly in vivo, inducing ICD within the tumor and activating endogenous dendritic cells in situ [271,272,273,274]. While this approach is technologically simpler and allows rapid immune activation, its efficacy depends strongly on the suppressive tumor microenvironment and the uniformity of nanoparticle accumulation. The development of nanoscale delivery systems that enable precise spatial, temporal, and dose-controlled co-administration of photosensitizers and immunotherapeutic agents represents a promising alternative to dendritic cell vaccines and may also serve as a complementary strategy.

Even when physical and pharmacological limitations are addressed, the biological efficacy of PDT remains constrained by tumor immunosuppression and resistance mechanisms—which is why combination treatment regimens have emerged as a promising research direction [275,276,277]. Undoubtedly, immunotherapeutic strategies currently represent the most promising direction. Combination regimens integrating PDT with immunotherapy are designed to enhance systemic antitumor immunity. The induction of immunological memory contributes to relapse prevention and represents a fundamental advantage over purely local tumor destruction approaches. Nevertheless, even optimally induced ICD by PDT is often insufficient for the eradication of aggressive cancer types (e.g., melanoma, sarcoma, pancreatic cancer) due to the strong immunosuppressive mechanisms of the tumor microenvironment and the low baseline immunogenicity of the tumor. In this context, combined approaches appear particularly promising.

The combination of photodynamic therapy with immune checkpoint inhibitors overcomes the main mechanism of resistance to inhibitors—namely, the immunologically “cold” tumor microenvironment. The key advantage of this strategy lies in its synergy: by inducing immunogenic cell death, PDT ensures the release of tumor antigens and DAMPs, which facilitate the recruitment of dendritic cells and cytotoxic T lymphocytes to the tumor site. PDT transforms the microenvironment into an immunologically “hot” state, whereas subsequent administration of anti-PD-1/PD-L1 antibodies or CTLA-4 blockers eliminates inhibitory signals from activated T cells, preventing their exhaustion and enhancing the systemic antitumor response [221,278]. Experimental evidence indicates that the reduction in distant metastases and the formation of immunological memory are observed exclusively when PDT is combined with immune checkpoint inhibitors, whereas monotherapy with either approach fails to achieve such an effect [279]. Nevertheless, this strategy inherits the fundamental limitations of PDT itself—pronounced oxygen dependence, limited light penetration depth, and systemic phototoxicity. Consequently, despite the evident necessity of combination with checkpoint inhibitors, the optimal regimen (sequence of administration and dosage) remains unresolved: an inadequately designed protocol can not only reduce the therapeutic effect but also trigger immunosuppression [278].

**Figure 3:**
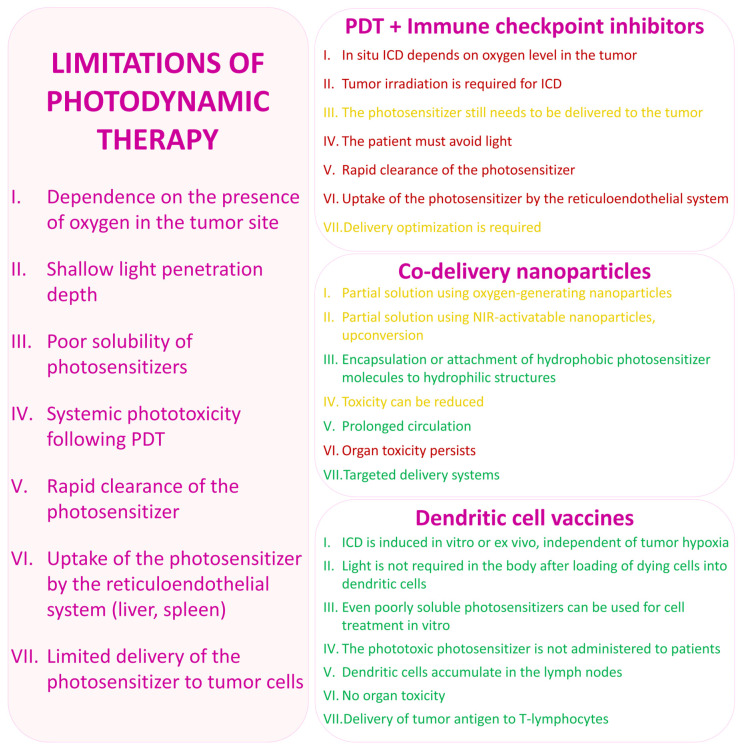
Comparison of three immunophotodynamic strategies: overcoming the limitations of classical PDT. The left panel lists the seven fundamental limitations of classical PDT. The three right panels describe how each strategy—combination of PDT with immune checkpoint inhibitors, nanoparticle-based co-delivery systems, and dendritic cell vaccines—addresses these limitations. In our view, dendritic cell vaccines overcome most of the physicochemical limitations of PDT, whereas the combination of PDT with immune checkpoint inhibitors preserves them almost entirely. Nanoparticle-based co-delivery systems occupy an intermediate position, offering only partial solutions.

The integration of PDT with dendritic cell vaccination aligns well with the principles of personalized medicine: dendritic cells can be generated from the patient’s own monocytes and loaded with autologous tumor antigens (e.g., obtained from biopsy material or following neoadjuvant PDT), thereby ensuring high specificity and minimizing the risk of autoimmune reactions. Of particular interest are protocols aimed at activating antitumor immunity, including strategies for converting immunologically “cold” tumors into lesions characterized by pronounced immune cell infiltration (Figure 3).

However, it should be acknowledged that dendritic cell vaccination has certain limitations compared with other immunotherapeutic strategies, such as immune checkpoint inhibitors and CAR T-cell therapy. In the treatment of solid tumors (lung cancer, melanoma, renal cancer, etc.), immune checkpoint inhibitors (e.g., pembrolizumab, nivolumab) have gained considerable recognition: they “release the brakes” on the patient’s own T cells and produce durable responses in 15–45% of patients, although their efficacy depends on the presence of an immunologically “hot” tumor, and they are associated with the risk of severe immune-related adverse events (e.g., colitis, pneumonitis) [280]. In contrast, CAR T-cell therapy has demonstrated remarkable success in hematological malignancies but remains experimental in solid tumors (actual response rates <5–10%), as it is limited by the tumor microenvironment, the absence of a safe universal antigen, and the risk of severe cytokine release syndrome. Dendritic cell immunotherapy exhibits the most favorable safety profile, but current protocols remain insufficiently effective.

Although immunotherapy based on immune checkpoint inhibitors has revolutionized the clinical treatment of various malignancies, a substantial proportion of patients demonstrate resistance to this approach [281]. One of the key factors underlying resistance to immune checkpoint inhibitors is the initially low infiltration of the tumor microenvironment by cytotoxic T lymphocytes [282].

The application of dendritic cell therapy has the potential to enhance the efficacy of immune checkpoint inhibition. This hypothesis is supported by studies demonstrating the necessity of recruiting effector T cells into the tumor microenvironment [283]. Indeed, dendritic cell infiltration has been shown to correlate with the success of immunotherapy in cancer patients; therefore, the combination of dendritic cell therapy with immune checkpoint inhibitors is being actively investigated in clinical practice [284]. In several phase I/II clinical trials, the combination of dendritic cell vaccination with immune checkpoint inhibitors, particularly antibodies targeting CTLA-4 [285,286], has been evaluated. The results of these studies indicate that dendritic cell vaccination can enhance antigen-specific T-cell responses and improve clinical outcomes in certain cancer types.

Among the strategies discussed, the combination of dendritic cell vaccination with immune checkpoint inhibitors has achieved the greatest progress toward clinical efficacy. Early-phase studies have demonstrated an acceptable safety profile and pronounced immunological activity of this strategy. This appears to us to be a promising solution, although it does require the development of a high-tech cell-based product.

Thus, despite the differences in the implementation of immunotherapy—ranging from ex vivo dendritic cell activation to in vivo nanoparticle-based co-delivery—the key requirement for effective immunophotodynamic therapy remains the controlled induction of immunogenic cell death. Future developments will likely focus on hybrid platforms that combine the spatiotemporal precision of nanoscale delivery with a personalized antigenic composition.

From our perspective, this revised view of photodynamic therapy enables the full realization of its therapeutic potential: to combine local tumor destruction, systemic immune activation, and the establishment of immunological memory for personalized cancer treatment.

## Figures and Tables

**Table 1:** Characteristics of various forms of photoinduced regulated cell death.

Form of Cell Death	Cellular Morphology	Key Features of the Mechanism	Photosensitizers
**Apoptosis**	Cell shrinkage and reduction in size, chromatin condensation, nuclear fragmentation, formation of apoptotic bodies.	In the mitochondrial apoptotic pathway, the nuclear protein p53 activates pro-apoptotic regulators (BID/tBID) and inactivates anti-apoptotic proteins of the Bcl-2 family. This removes inhibition of the BAX/BAK effectors, leading to their oligomerization, pore formation in the outer mitochondrial membrane, and release of apoptotic factors (cytochrome c, procaspase-9, APAF-1) into the cytosol. There, an apoptosome is formed, which activates procaspase-9. Activated caspase-9 subsequently cleaves procaspases-3 and -7, initiating the terminal caspase cascade.	Photofrin [92], hypericin [107], Rose Bengal acetate [108], protoporphyrin IX induced by exogenous 5-aminolevulinic acid (5-ALA) [109], 2-(1-Hexyloxyethyl)-2-devinyl pyropheophorbide-a, 8-methoxypsoralen [110], meso-tetrakis (4-carboxyphenyl) porphyrin sodium salt, Zn-meso-tetrakis (4-carboxyphenyl) porphyrin sodium salt [111]
**Autophagy**	Double-membrane structures, autophagosomes, are formed in the cell. Individual organelles are engulfed, autophagosomes fuse with lysosomes, and cellular structures are degraded by hydrolases.	Induction of cell death activates macroautophagy signaling pathways involving proteins derived from damaged mitochondria and the endoplasmic reticulum. Ubiquitin-dependent labeling of damaged components recruits receptors (e.g., p62), which link substrates to the forming autophagosome. The resulting double-membrane autophagosome engulfs damaged organelles and subsequently fuses with the lysosome, forming an autolysosome. Morphologically, the process is characterized by cytoplasmic vacuolization and the presence of autophagosomes and autolysosomes. Assembly and elongation of the autophagosome membrane are regulated by the PI3K complex (Beclin-1, Vps34), ubiquitin-like conjugation systems (Atg12–Atg5–Atg16L1), as well as LC3-II and the Atg4 protease involved in its processing and recycling.	Sodium sinoporphyrin [112], verteporfin [113], CPO [112], hypericin [114], temoporfin [113]
**Necroptosis**	Increased plasma membrane permeability, increased cell volume, swelling of organelles, followed by cell lysis.	Necroptosis is regulated by the kinases RIPK1, RIPK3, and the pseudokinase MLKL. Upon binding of TNF-α to TNFR1, complex I is formed, where polyubiquitination of RIPK1 activates NF-κB and promotes cell survival. Disruption of RIPK1 ubiquitination leads to the formation of complex II (the ripoptosome), in which RIPK1 interacts with RIPK3 via RHIM motifs to form the necrosome. Upon inhibition of caspase-8, RIPK3 phosphorylates MLKL, inducing its oligomerization and translocation to the membrane, where it forms cation-selective pores, resulting in osmotic cell lysis.	Protoporphyrin IX induced by exogenous 5-aminolevulinic acid (5-ALA) [115]
**Paraptosis**	Appearance of multiple vacuoles gradually filling the cytoplasm; swelling of the endoplasmic reticulum and mitochondria.	The precise mechanism of paraptosis remains under investigation. Induction of paraptosis is associated with IGF1R overexpression, proteasome inhibition, ER stress, and oxidative stress. Specific stimuli activate RIP1 kinase and promote the accumulation of misfolded proteins, leading to maladaptive ER stress. This process induces opening of IP3 receptors and massive release of Ca^2+^ from the ER, resulting in cytoskeletal hypercontraction and mitochondrial calcium overload. Mitochondria undergo swelling due to opening of the mPTP without cytochrome c release, while AIF translocates to the nucleus, initiating DNA fragmentation.	m-TSX, p-TSX [116,117], verteporfin [103]
**Ferroptosis**	Loss of mitochondrial cristae and mitochondrial shrinkage; increased permeability of the plasma membrane	Iron-dependent regulated cell death associated with intracellular accumulation of ROS generated via the Fenton reaction, leading to excessive oxidation of membrane lipids.	Photosens, 5-ALA, talaporfin sodium (TS-PDT) [118], two cyclometallated Ir(III) complexes (IrL1 and MitoIrL2) [119], chlorin e6 [120,121]
**Parthanatos**	DNA fragmentation, nuclear pyknosis, necrotic membrane changes	Parthanatos is characterized by hyperactivation of PARP1 (poly(ADP-ribose) polymerase 1), a protein involved in the cellular response to DNA damage. Hyperactivation of PARP1 leads to depletion of NAD^+^ and ATP and loss of mitochondrial transmembrane potential.	Na-H_2_TCPP [111], Deoxypodophyllotoxin [122]
**Pyroptosis**	In early stages, characterized by chromatin condensation and DNA fragmentation, similar to apoptosis; in later stages, disruption of the plasma membrane, release of cellular contents into the extracellular environment, and induction of inflammatory processes in the tissue	Pyroptosis is initiated by cleavage of GSDM (Gasdermin) by caspase-1 and, in the non-canonical pathway, by caspases 4/5/11. This results in the formation of the GSDM-N fragment, which forms pores in the plasma membrane and leads to cell lysis. Caspase-1 simultaneously activates proinflammatory cytokines, including IL-1β and IL-18, which are released through GSDM-N pores.	NPe6 [123], verteporfin (BPD) [124], hypericin [125], 1,1,2,2-tetrachloroethane, diphenylmethylenemalononitrile [126]
**Cuproptosis**	Reduction in mitochondrial volume (shrinkage), appearance of cytoplasmic vacuoles, destruction of the endoplasmic reticulum, and loss of chromatin structure.	The mechanisms are not fully understood. Excessive copper accumulation initiates ROS production via copper-dependent Fenton reactions and simultaneously leads to destabilization of Fe-S clusters.	Aloe emodin (AE) loaded with copper ions (Cu), and self-assembled into nanoparticles (NPs) under the modification of PEG2k-DSPE-FA [127]

## Data Availability

No new data were created or analyzed in this study. Data sharing is not applicable.

## Supplementary Materials

The following supporting information can be downloaded at: https://www.mdpi.com/article/10.3390/pharmaceutics18050588/s1, Table S1: Clinically approved photosensitizers. References [287,288,289,290,291,292,293,294,295,296,297,298,299,300,301,302,303,304,305,306,307,308,309,310,311,312,313,314,315] cited in Supplementary Materials.

## Abbreviations

The following abbreviations are used in this manuscript:

5-ALA	5-Aminolevulinic acid
ABC	ATP-binding cassette
ABCB1	Multidrug resistance protein 1
ABCG2	ATP-binding cassette subfamily G member 2
AIF	Apoptosis-inducing factor
AIM2	Absent in melanoma 2
AIP1/Alix	ALG-2-interacting protein 1 (programmed cell death 6-interacting protein, PDCD6IP)
AlPcS_2_a	Aluminum phthalocyanine disulfonate, isomer a
ANXA	Annexin A protein family (e.g., Annexin A1, Annexin A2)
APE1	Apurinic/apyrimidinic endonuclease 1
AP-1	Activator protein 1
APAF-1	Apoptotic protease-activating factor 1
ATP	Adenosine triphosphate
ATF4	Activating transcription factor 4
ATF6	Activating transcription factor 6
ASC	Apoptosis-associated speck-like protein containing a CARD
Atg4	Autophagy-related protein 4
Atg12	Autophagy-related protein 12
Atg16L1	Autophagy-related protein 16-like 1
Bak	Bcl-2 homologous antagonist/killer
Bax	Bcl-2-associated X protein
Bcl-2	B-cell lymphoma 2
Bcl-B	Bcl-2-related protein A1
Bcl-w	Bcl-2-like protein 2
Bcl-xL	B-cell lymphoma-extra large
BER	Base excision repair
Bfl-1	Bcl-2-related protein A1
BID	BH3-interacting domain death agonist
Bok	Bcl-2-related ovarian killer
BPD	Benzoporphyrin derivative
BPD-MA	Benzoporphyrin derivative monoacid ring A
Ca^2+^	Calcium
CCPS	Chimeric cross-linked polymersome
CLEC9A	C-type lectin domain family 9 member A
CPO	Chlorin p6 (or a related chlorin-based photosensitizer)
CRT	Calreticulin
CSF-1R	Colony-stimulating factor 1 receptor
CTL	Cytotoxic T lymphocyte
CTLA-4	Cytotoxic T-lymphocyte associated protein 4
CuO	Copper(II) oxide
Cu,Zn-SOD	Copper/zinc-dependent superoxide dismutase (SOD1)
DAMPs	Damage-associated molecular patterns
DC	Dendritic cell
DLAT	Dihydrolipoamide S-acetyltransferase
DNA	Deoxyribonucleic acid
eIF2α	Eukaryotic translation initiation factor 2 alpha
ER	Endoplasmic reticulum
ER stress	Endoplasmic reticulum stress
ERK2	Extracellular signal-regulated kinase 2
FDA	Food and Drug Administration
Fas	Fas cell surface death receptor (CD95)
GSDM	Gasdermin
GSDM-D	Gasdermin-D
GSDM-N	N-terminal domain of gasdermin family proteins
GSH	Glutathione
GPX4	Glutathione peroxidase 4
GST	Glutathione S-transferase
GRP78	Glucose-regulated protein 78 kDa
H2O2	Hydrogen peroxide
HIF-1	Hypoxia-inducible factor 1
HMGB1	High mobility group box 1 protein
HO_2_•	Hydroperoxide radical
HPPH	2-(1-Hexyloxyethyl)-2-devinyl pyropheophorbide-a
HpD	Hematoporphyrin derivative
HSF1	Heat shock factor 1
HSP	Heat shock protein
HSP27	Heat shock protein 27
HSP60	Heat shock protein 60
HSP70	Heat shock protein 70
ICD	Immunogenic cell death
IFN-γ	Interferon-gamma
IGF1R	Insulin-like growth factor 1 receptor
IL-1β	Interleukin-1 beta
IL-6	Interleukin-6
IL-10	Interleukin-10
IL-12	Interleukin-12
IL-18	Interleukin-18
IRE1	Inositol-requiring enzyme 1
IP3	Inositol trisphosphate
JNK	c-Jun N-terminal kinase
Keap1	Kelch-like ECH-associated protein 1
LC3	Microtubule-associated protein 1A/1B light chain 3
LPS	Lipopolysaccharide
MAPK	Mitogen-activated protein kinase
MC38	Murine colon adenocarcinoma cell line
Mcl-1	Myeloid cell leukemia 1
MDR1	Multidrug resistance protein 1 (ABCB1)
MSNs	Mesoporous ones
MHC-II	Major histocompatibility complex class II
MIF	Macrophage migration inhibitory factor
MnSOD	Manganese-dependent superoxide dismutase (SOD2)
m-THPC	meta-Tetra(hydroxyphenyl)chlorin (Temoporfin)
m-TSX	meta-TSX (porphyrin-based photosensitizer, TSX family)
mTORC1	Mechanistic (mammalian) target of rapamycin complex 1
NAD^+^	Nicotinamide adenine dinucleotide (oxidized form)
NCCD	Nomenclature Committee on Cell Death
NF-κB	Nuclear factor kappa B
NIR	Near-infrared
NK cells	Natural killer cells
NLRP1	NLR family pyrin domain-containing 1
NLRP3	NLR family pyrin domain-containing 3
NPe6	Talaporfin sodium (mono-L-aspartyl chlorin e6)
NO	Nitric oxide
Nrf2	Nuclear factor erythroid 2-related factor 2
O_2_	Molecular oxygen
O_2_^−^•	Superoxide anion radical
^1^O_2_	Singlet oxygen
OH•	Hydroxyl radical
Oxy-PDT	Oxygen-enhanced photodynamic therapy
PAMPs	Pathogen-associated molecular patterns
PARP1	Poly(ADP-ribose) polymerase 1
Pc 4	Phthalocyanine 4 (silicon phthalocyanine-based photosensitizer)
PD-1	Programmed cell death protein 1
PD-L1	Programmed death-ligand 1
PDT	Photodynamic therapy
PEG2k-DSPE-FA	Poly(ethylene glycol)-2000-distearoylphosphatidylethanolamine-folate
PERK	PKR-like ER kinase
PI3K	Phosphoinositide 3-kinase
^1^PS*	Excited singlet state
^3^PS*	Triplet state
PRRs	Pattern recognition receptors
PS	Photosensitizers
PTT	Photothermal therapy
RCD	Regulated cell death
RHIM	RIP homotypic interaction motif
RIPK1	Receptor-interacting protein kinase 1
RIPK3	Receptor-interacting protein kinase 3
ROS	Reactive oxygen species
SOD1	Copper/zinc-dependent superoxide dismutase 1
SOD2	Manganese-dependent superoxide dismutase 2
STAT3	Signal transducer and activator of transcription 3
TAA	Tumor-associated antigen
TCA	Tricarboxylic acid cycle (citric acid cycle)
TLR3	Toll-like receptor 3
TLR4	Toll-like receptor 4
TME	Tumor microenvironment
TNF-α	Tumor necrosis factor alpha
TNFR1	Tumor necrosis factor receptor 1
TORC1	Target of rapamycin complex 1
TRADD	TNF receptor-associated death domain protein
TRAF2	TNF receptor-associated factor 2
TS-PDT	Talaporfin sodium photodynamic therapy
TSA	Tumor-specific antigen
UPR	Unfolded protein response
VEGF	Vascular endothelial growth factor
Vps34	Vacuolar protein sorting 34 (class III phosphatidylinositol 3-kinase)
XBP1	X-box binding protein 1
XCR1	X-C motif chemokine receptor 1
ZBP1	Z-DNA binding protein 1 (Z-NA binding protein 1/DAI/DLM-1)
ZnO	Zinc oxide
cDC1	Conventional dendritic cells type 1
cIAP1/2	Cellular inhibitor of apoptosis protein 1 and 2
c-fos	Cellular fos proto-oncogene
c-jun	Cellular jun proto-oncogene
p-TSX	para-TSX (porphyrin-based photosensitizer, TSX family)
p53	Tumor protein p53
tBID	Truncated BH3-interacting domain death agonist
