# Supplementary material for: Photo- and Immunotherapy Interface: Can Dendritic Cell Vaccines Overcome the Limitations of PDT?
Source: Pharmaceutics. 2026 May 10;18(5):588. doi: 10.3390/pharmaceutics18050588 (PMC13210220; doi:10.3390/pharmaceutics18050588)
Supplement: Supplementary file 1 [file pharmaceutics-18-00588-s001.zip › pharmaceutics-4280106-supplementary.pdf]

Table S1. Clinically approved photosensitizers.

Clinical name or chemical name	Chemical structure	Photosensitizer manufacturer	In which countries is it approved?	Clinical application	References
First-generation photosensitizers					
hematoporphyrin derivative (HpD)	Photofrin	Axcan Pharma Inc	All over the world	non-small cell lung cancer, stomach cancer, bladder cancer, esophageal cancer, cervical cancer, brain cancer, ovarian cancer, etc.	[1,2,3,4]
	Photogem	State Research Center of Laser Medicine and P.A. Herzen Moscow Oncology Research Institute	Russia	skin cancer, intradermal metastases of breast cancer and melanoma, cancer of the tongue and oral mucosa, etc.	[5]
	Photosan		Germany	Head and neck tumors, pancreatic cancer, glioma, etc.	[6,7,8]
Second-generation photosensitizers					
Photosense	phthalocyanine	Federal State Unitary Enterprise "State Scientific Center "NIOPIK"	Russia	skin cancer, stomach cancer, early vulvar cancer, breast cancer, laryngeal cancer, dysplasia and early cervical cancer, etc.	[9,10]
Chlorines	Photoditazine	LLC "VETA-GRAND"	Russia	skin cancer (squamous cell, basal cell), lung cancer, etc.	[11,12]
	Radachlorin	RADA-PHARMA LLC	Russia, South Korea	superficial tumors of the skin and mucous membranes, primary multiple skin cancer, cervical cancer, etc.	[13]
	Foscan (mTHPC)	Biolitec	European Union, Norway, Iceland	head and neck cancer	[14]
	Photolon	RUE Belmedpreparaty	Republic of Belarus, Russia	fluorescence diagnostics and photodynamic therapy of skin cancer, disseminated metastases of melanoma and breast cancer, cancer of the mucous membranes	[15,16]

				(vulva, esophagus, rectum, etc.), precancerous diseases of the cervix, etc.	
	NPc6, MACE (mono-L-aspartyl-chlorin e 6),	«Meiji Seika Kaisha Ltd.» and «Sciences Corporation»	Japan, USA	skin cancer, lung cancer, head and neck tumors, etc.	[17]
	Photochlor, HPPH	Roswell Park Cancer Institute Medkoo Biosciences	USA	esophageal cancer	[18,19,20]
Purlytin	etiopurpurin selenium		USA	cutaneous metastases of breast cancer and Kaposi's sarcoma in HIV-infected patients	[21,22]
Lutex	lutetium texaphyrin		USA	metastases of breast cancer, melanoma, Kaposi's sarcoma, basal cell and squamous cell skin cancer	[2,22]
benzoporphyrin derivative	Visudyne	Novartis	Russia	retinal vascular neoplasms caused by age-related degenerative changes	[23]
	Verteporfin		China, Norway	skin cancer, psoriasis, basal cell carcinoma, age-related macular degeneration	[24]
5-aminolevulinic acid protoporphyrin IX	Alasens	Federal State Unitary Enterprise "State Scientific Center "NIOPIK"	Russia	Early endometrial cancer, precancerous and tumorous pathology of the cervix, metastatic peritoneal lesions fluorescence diagnostics of malignant neoplasms of the bladder, larynx, trachea, bronchi, oral mucosa, and gastrointestinal tract	[5,25]
	Levulan	DUSA Fudan-Zhangjiang	USA, China, European Union	mild to moderate actinic keratosis	[4,26]
	Metvix	Photocure ASA	European Union, USA, Canada	basal cell carcinoma, actinic keratosis	[27,28,29]

Tookad	Bacteriochlorophyll padeliporphin	Steba Biotech Negma-Lerads	Europe, Israel, Mexico	prostate cancer	[22,30]

1. Gunaydin, G.; Gedik, M.E.; Ayan, S. Photodynamic Therapy for the Treatment and Diagnosis of Cancer–A Review of the Current Clinical Status. *Front. Chem.* **2021**, *9*, doi:10.3389/fchem.2021.686303.
2. Dougherty, T.J.; Gomer, C.J.; Henderson, B.W.; Jori, G.; Kessel, D.; Korbelik, M.; Moan, J.; Peng, Q. Photodynamic Therapy. *JNCI Journal of the National Cancer Institute* **1998**, *90*, 889–905, doi:10.1093/jnci/90.12.889.
3. Agostinis, P.; Berg, K.; Cengel, K.A.; Foster, T.H.; Girotti, A.W.; Gollnick, S.O.; Hahn, S.M.; Hamblin, M.R.; Juzeniene, A.; Kessel, D.; et al. Photodynamic Therapy of Cancer: An Update. *CA Cancer J. Clin.* **2011**, *61*, 250–281, doi:10.3322/caac.20114.
4. Xiao, Q.; Wu, J.; Pang, X.; Jiang, Y.; Wang, P.; Leung, A.W.; Gao, L.; Jiang, S.; Xu, C. Discovery and Development of Natural Products and Their Derivatives as Photosensitizers for Photodynamic Therapy. *Curr. Med. Chem.* **2018**, *25*, 839–860, doi:10.2174/0929867324666170823143137.
5. Stranadko, E.F. Main Stages of Development of Photodynamic Therapy in Russia. *Biomedical Photonics* **2015**, *4*, 3–10, doi:10.24931/2413-9432-2015-4-1-3-10.
6. Allison, R.R.; Cuenca, R.E.; Downie, G.H.; Camnitz, P.; Brodish, B.; Sibata, C.H. Clinical Photodynamic Therapy of Head and Neck Cancers—A Review of Applications and Outcomes. *Photodiagnosis Photodyn. Ther.* **2005**, *2*, 205–222, doi:10.1016/S1572-1000(05)00092-X.
7. Gallardo-Villagrán, M.; Leger, D.Y.; Liagre, B.; Therrien, B. Photosensitizers Used in the Photodynamic Therapy of Rheumatoid Arthritis. *Int. J. Mol. Sci.* **2019**, *20*, 3339, doi:10.3390/ijms20133339.
8. Yu, Z.; Li, H.; Zhang, L.-M.; Zhu, Z.; Yang, L. Enhancement of Phototoxicity against Human Pancreatic Cancer Cells with Photosensitizer-Encapsulated Amphiphilic Sodium Alginate Derivative Nanoparticles. *Int. J. Pharm.* **2014**, *473*, 501–509, doi:10.1016/j.ijpharm.2014.07.046.
9. Sokolov, V.V.; C.V.I.; Y.R.I.; A.E.; F.E.; B.T.; V.G.N.; Z.N.N.; S.V.; Z.M.B. Photodynamic Therapy (PDT) of Malignant Tumors by Photosensitizer Photosens: Results of 45 Clinical Cases. In Proceedings of the BiOSEurope'95; International Society for Optics and Photonics, 1996; pp. 281–287.
10. O'Connor, A.E.; Gallagher, W.M.; Byrne, A.T. Porphyrin and Nonporphyrin Photosensitizers in Oncology: Preclinical and Clinical Advances in Photodynamic Therapy. *Photochem. Photobiol.* **2009**, *85*, 1053–1074, doi:10.1111/j.1751-1097.2009.00585.x.
11. Kaplan, M.A.K.V.N.R.Y.S.Y.-I.E. V. Photoditazin: An Effective Photosensitizer for Photodynamic Therapy. *Russian Biotherapeutic Journal* **2004**, *3*, 50.
12. Filonenko, E.V.S.L.G. Photodynamic Therapy in Clinical Practice. *Biomedical Photonics* **2016**, *5*, 26–37.
13. Filonenko, E. V.; Serova, L.G.; Ivanova-Radkevich, V.I. Results from Phase III Clinical Trials with Radachlorine for Photodynamic Therapy of Pre-Cancer and Early Cancer of Cervix. *Biomedical Photonics* **2015**, *4*, 36–42, doi:10.24931/2413-9432-2015-4-3-36-42.
14. Uprety, B.; Abrahamse, H. Semiconductor Quantum Dots for Photodynamic Therapy: Recent Advances. *Front. Chem.* **2022**, *10*, doi:10.3389/fchem.2022.946574.
15. Kostenich, G.A.; Zhuravkin, I.N.; Zhavrid, E.A. Experimental Grounds for Using Chlorin P6 in the Photodynamic Therapy of Malignant Tumors. *J. Photochem. Photobiol. B* **1994**, *22*, 211–217, doi:10.1016/1011-1344(93)06974-8.
16. Petrov, P.T.T.T.V.I.G.A.S.S.V.M.L.N.I.Yu.P. Photolon: A Drug for Photodynamic Therapy and Diagnostics. Clinical Application Experience. *Vestnik Farmatsii* **2007**, 69–81.
17. Deda, D.K.; Araki, K. Nanotechnology, Light and Chemical Action: An Effective Combination to Kill Cancer Cells. *J. Braz. Chem. Soc.* **2015**, doi:10.5935/0103-5053.20150316.
18. Allison, R.R.; Sibata, C.H. Oncologic Photodynamic Therapy Photosensitizers: A Clinical Review. *Photodiagnosis Photodyn. Ther.* **2010**, *7*, 61–75, doi:10.1016/j.pdpdt.2010.02.001.

19. PANDEY, R.K.; BELLNIER, D.A.; SMITH, K.M.; DOUGHERTY, T.J. CHLORIN AND PORPHYRIN DERIVATIVES AS POTENTIAL PHOTOSENSITIZERS IN PHOTODYNAMIC THERAPY. *Photochem. Photobiol.* **1991**, *53*, 65–72, doi:10.1111/j.1751-1097.1991.tb08468.x.
20. Yoon, I.; Li, J.Z.; Shim, Y.K. Advance in Photosensitizers and Light Delivery for Photodynamic Therapy. *Clin. Endosc.* **2013**, *46*, 7, doi:10.5946/ce.2013.46.1.7.
21. Kaplan, M.J.; Somers, R.G.; Greenberg, R.H.; Ackler, J. Photodynamic Therapy in the Management of Metastatic Cutaneous Adenocarcinomas: Case Reports from Phase 1/2 Studies Using Tin Ethyl Etiopurpurin (SnET2). *J. Surg. Oncol.* **1998**, *67*, 121–125, doi:10.1002/(SICI)1096-9098(199802)67:2<121::AID-JSO9>3.0.CO;2-C.
22. Karges, J. Clinical Development of Metal Complexes as Photosensitizers for Photodynamic Therapy of Cancer. *Angew. Chem. Int. Ed.* **2022**, *61*, doi:10.1002/anie.202112236.
23. Baskaran, R.; Lee, J.; Yang, S.-G. Clinical Development of Photodynamic Agents and Therapeutic Applications. *Biomater. Res.* **2018**, *22*, doi:10.1186/s40824-018-0140-z.
24. Mellish, K.J.; Brown, S.B. Verteporfin: A Milestone in Opthalmology and Photodynamic Therapy. *Expert Opin. Pharmacother.* **2001**, *2*, 351–361, doi:10.1517/14656566.2.2.351.
25. Filonenko, E. V. Fluorescence Diagnosis with Alasens in Patients with Skin Cancer. *Biomedical Photonics* **2015**, *4*, 14–17.
26. Peng, Q.; Berg, K.; Moan, J.; Kongshaug, M.; Nesland, J.M. 5-Aminolevulinic Acid-Based Photodynamic Therapy: Principles and Experimental Research. *Photochem. Photobiol.* **1997**, *65*, 235–251, doi:10.1111/j.1751-1097.1997.tb08549.x.
27. Calzavara-Pinton, P.; Venturini, M.; Sala, R. Photodynamic Therapy: Update 2006 Part 2: Clinical Results. *Journal of the European Academy of Dermatology and Venereology* **2007**, *21*, 439–451, doi:10.1111/j.1468-3083.2006.02038.x.
28. Espiñeira Sicre, J.; García Sirvent, L.; Ruiz Sánchez, J.; García Fernández, L.; Soro Martínez, P.; Miralles Botella, J.; Fernández Fornos, L.; Onrubia Pintado, J.A.; Cuesta Montero, L. Neoadjuvant Photodynamic Therapy as a Therapeutic Alternative in Multiple Basal Cell Carcinoma Induced by Radiotherapy. *Photodiagnosis Photodyn. Ther.* **2023**, *44*, 103820, doi:10.1016/j.pdpdt.2023.103820.
29. Zeitouni, N.C.O.A.N.D.J. Photodynamic Therapy. In *Skin Cancer Management*; Springer: New York, 2009; pp. 41–56.
30. Vale, N.; Ramos, R.; Cruz, I.; Pereira, M. Application of Peptide-Conjugated Photosensitizers for Photodynamic Cancer Therapy: A Review. *Organics* **2024**, *5*, 429–442, doi:10.3390/org5040022.
